# Structure‐Binding Relationship of 2‐Amino‐1,8‐Naphthyridine Dimers: Role of Linkage Positions on DNA and RNA Recognition

**DOI:** 10.1002/chem.202500425

**Published:** 2025-04-10

**Authors:** Bimolendu Das, Satoki Kuwahara, Ryosuke Ishimaru, Eitaro Murakami, Yasue Harada, Kazuhiko Nakatani

**Affiliations:** ^1^ Sanken The University of Osaka 8–1 Mihogaoka Ibaraki 567-0047 Japan

**Keywords:** small molecule, DANN, RNA, Recognition, naphthyridine

## Abstract

The study explores the synthesis, structural analysis, and binding properties of eight analogs of 2‐amino‐1,8‐naphthyridine dimers (ANPxys) targeting DNA and RNA. These dimers, derived from ANP77, are connected at varying positions to investigate how positional alterations influence molecular conformations and their interactions with nucleic acids. The primary focus lies on evaluating the effects of these structural variations on DNA and RNA binding through fluorescence quenching and thermal denaturation assays. Absorption and fluorescence measurements revealed distinct electronic states for ANPxys, with emission maxima between 389.5 and 398.5 nm. Conformational analysis indicated that most ANPxys adopt unstacked conformations in aqueous solutions, though some, like ANP47 and ANP67, showed higher probabilities of stacked conformations. Thermal denaturation studies demonstrated ANPxys bind and stabilize cytosine‐rich DNA motifs with varying affinities, with ANP77 showing the strongest effects. RNA binding studies targeting U/CC motifs across 256 sequences revealed unique fluorescence quenching patterns for each ANPxy, reflecting sequence specificity. Hierarchical clustering grouped ANPxys into parallel‐stacked and twisted‐stacked clusters, correlating their conformations with binding preferences. This work highlights the critical role of connection positions in determining ANPxy binding specificity and conformational behavior. The findings provide a basis for designing small molecules with tunable structures and enhanced RNA‐binding capabilities, paving the way for the development of RNA‐targeted therapeutics.

## Graphical abstract

Altering the connecting positions of two 2‐amino‐1,8‐naphthyridine units modulates binding to distinct DNA and RNA motifs. This structural variation influences molecular interactions, affecting stacking tendencies and hydrogen bonding. The positional differences determine how these molecules recognize cytosine‐rich sequences, impacting their ability to stabilize nucleic acid structures and paving the way for designing RNA‐targeted small molecules.

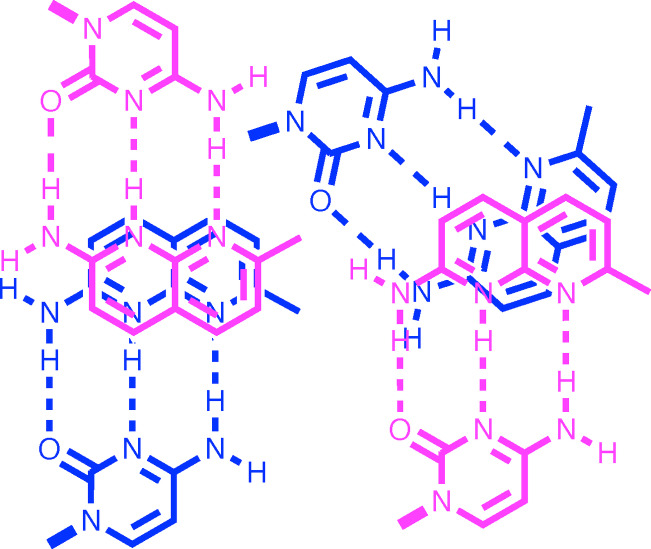

## Introduction

Stacking of aromatic molecules in aqueous environments is a fundamental interaction in the formation of tertiary structures and molecular recognition events involving nucleic acids.[^1^, ^2^, ^3^] B‐form DNA duplexes consist of continuous stacks of A−T and G−C base pairs, forming a hydrophobic core surrounded by a hydrophilic sugar‐phosphate backbone. Stacked base pairs exhibit properties distinct from those of the original base pairs, particularly in their electronic states.^[4]^ For example, guanine stacked with an adjacent guanine in double‐stranded DNA (dsDNA) has a higher HOMO level compared to a single guanine and acts as a hole‐trapping site in DNA‐mediated hole transport.[^5^, ^6^] The orientation of two guanines significantly affects the electronic states of guanine doublets.^[7]^ In a B‐form duplex, the specific orientation of two guanines is determined by the conformation of the sugar‐phosphate backbone, allowing the HOMO to localize at the 5′‐side guanine. The influence of aromatic molecule orientation is also demonstrated in the molecular recognition of DNA by pyrrole‐imidazole polyamides (PIPs).^[8]^ Two PIPs stacked in the minor groove of a B‐form duplex arrange hydrogen bonding groups necessary for distinguishing A−T from G−C base pairs, achieving high sequence specificity for the binding.^[9]^ In other words, the precise spatial arrangement of two PIPs is driven by stacking and hydrogen bonding with the floor of the minor groove, and such arrangement is facilitated by linking the two PIPs with a designated amide linker.

**Figure 1:**
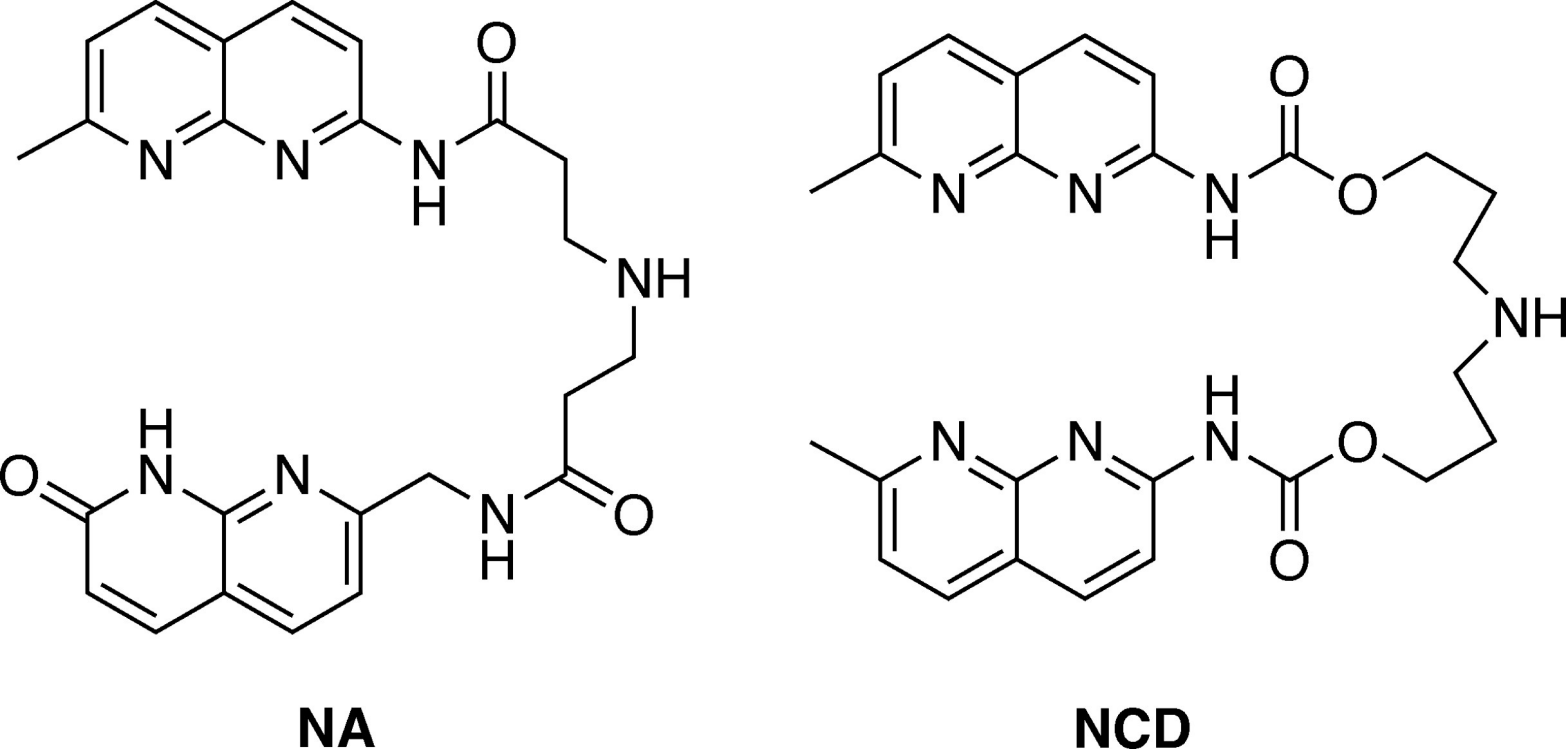
Structures of NA and NCD.

Last two decades, small molecules targeting DNA and RNA significantly increased the importance as the therapeutic drugs,[^10^, ^11^, ^12^, ^13^, ^14^, ^15^, ^16^, ^17^, ^18^, ^19^, ^20^, ^21^] and those binding to DNA and RNA with sequence and structure selectivity are the candidates.^[8]^ We studied mismatch‐binding ligands (MBLs) consisted of two aromatic heterocycles possessing hydrogen bonding groups at their molecular edges and a linker connecting them.^[22]^ One of MBLs, NA molecule recognized the A−A mismatched base pairs flanked by two C−G base pairs (CAG/CAG),^[23]^ whereas NCD, another example of MBLs recognized the G−G mismatched base pair flanked by two C−G base pairs (CGG/CGG).^[24]^ (Figure 1) Our studies on the structural analyses of NA bound to the CAG/CAG site and NCD bound to the CGG/CGG site demonstrated that two NA and NCD molecules were spontaneously assembled at the CAG/CAG and CGG/CGG site, respectively, with each aromatic heterocycle being stacked with the other heterocycle and neighboring base pairs, and positioned itself facing the nucleic acid bases. Subsequent studies on the structure‐binding activity of NA revealed that the chemical structure of the linker connecting two aromatic heterocycles significantly modulates the binding efficiency, likely due to constraints on the spatial orientation of the heterocycles.^[25]^

**Figure 2:**
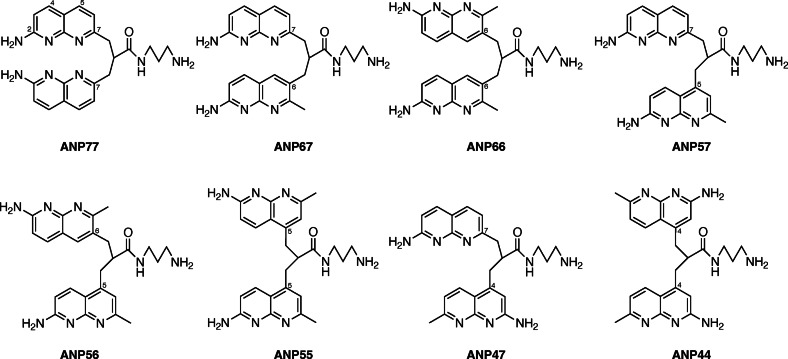
ANP77 and seven analogues ANPxy.

One of the heterocycles used for MBLs, 2‐acylamino‐1,8‐naphthyridine features a hydrogen bonding donor (D) of N−H and two acceptors (A) of ring nitrogen, with the alignment D−A‐A. This alignment is complementary to that of guanine (A−D‐D). Interestingly, 2‐amino‐1,8‐naphthyridine binds to cytosine rather than guanine, due to protonation at the ring nitrogen at a near neutral pH, which creates a D−D‐A alignment of hydrogen bonding groups, complementary to that of cytosine (A−A‐D). We synthesized dimers of 2‐amino‐1,8‐naphthyridine connected at the amino group or at C7 with different linkers. These studies revealed that the molecules exhibited varying binding affinities for cytosine‐rich sequences. Among these, ANP77 (Figure 2), with two 2‐amino‐1,8‐naphthyridines connected by a three‐atom linker at C7, showed high affinity for C/CC and T/CC motifs in dsDNA.[^26^, ^27^] To further explore ANP77′s potential as a DNA and RNA binding ligand, we examined its binding to RNAs containing C/CC and U/CC motifs flanked by all possible combinations of match and mismatch base pairs with fluorometric assay.^[26]^ The fluorescence quenching pattern analyzed by a heat map of 256 RNAs (N_1_UN_2_/N_3_CCN_4_, where N represents any one of four nucleotide bases) showed that ANP77 fluorescence was efficiently quenched with the sequence involving cytosines at N_2_ and N_3_ positions. While two 2‐amino‐1,8‐naphthyridines in ANP77 were connected both at C7 position, the heterocycle can be connected at different positions. Altering the connecting position can significantly affect the conformation, spatial arrangement, and orientation of the heterocycles, and, hence, the binding efficiency and selectivity. Here, we report the synthesis of seven ANP77 analogues with altered connection sites and investigate their binding characteristics to the U/CC motif RNA with 256 different flanking base combinations by fluorometric analysis.

### Design and Synthesis of ANPxy

**Scheme 1:**
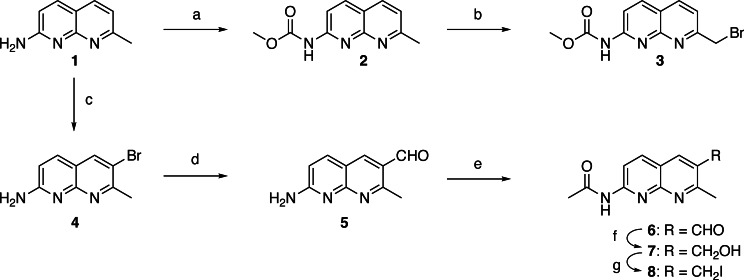
(a) Methyl chloroformate, K_2_CO_3_, acetone, 0 °C to r.t., overnight, 64 %; (b) NBS, BPO, CHCl_3_/CH_3_CN (5 : 4), reflux, 1 h, 31 %; (c) Br_2_, 1,4‐dioxane/water (1 : 1), 0 °C, 1 h, r.t., 3.5 h, 66 %, (d) *n*‐BuLi, THF, –100 °C, 1 h, then DMF, THF, –100 °C, 2 h; (e) Ac_2_O, Py, 70 °C, 2 h; (f) NaBH_4_, EtOH, 0 °C to r.t., 1 h, 35 % for 3 steps; (g) MsCl, Et_3_N, CH_2_Cl_2_, 0 °C to r.t., 3 h, then NaI, acetone, reflux, 2 h, 49 % for 2 steps.

**Scheme 2:**
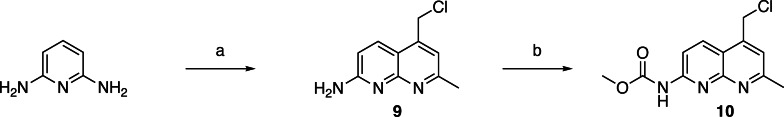
(a) 1‐chloro‐2,4‐pentandione, 85 % H_3_PO_4_, 80 °C, 20 h, 15 %; (b) Methyl chloroformate, K_2_CO_3_, r.t., 61 h, 93 %.

**Scheme 3:**
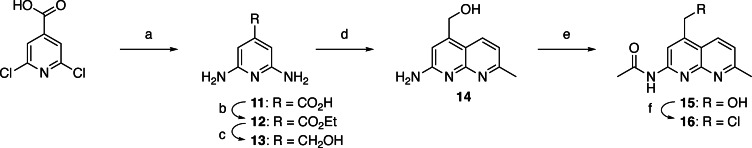
(a) 28 % NH_4_OH, Cu, 180 °C (sealed tube), 20.5 h; (b) 4 M HCl, EtOAc, EtOH, 80 °C, 18 h, 38 % for 2 steps; (c) LiAlH_4_, THF, 0 °C to r.t., 3 h, 86 %; (d) 4,4‐dimethoxybutan‐2‐one, 85 % H_3_PO_4_, 90 °C, 15 h, 18 %; (e) Ac_2_O, 60 °C, 3.5 h, then K_2_CO_3_, MeOH, r.t., 1.5 h, 55 %; (f) SOCl_2_, r.t., 2 h, 47 %.

**Scheme 4:**
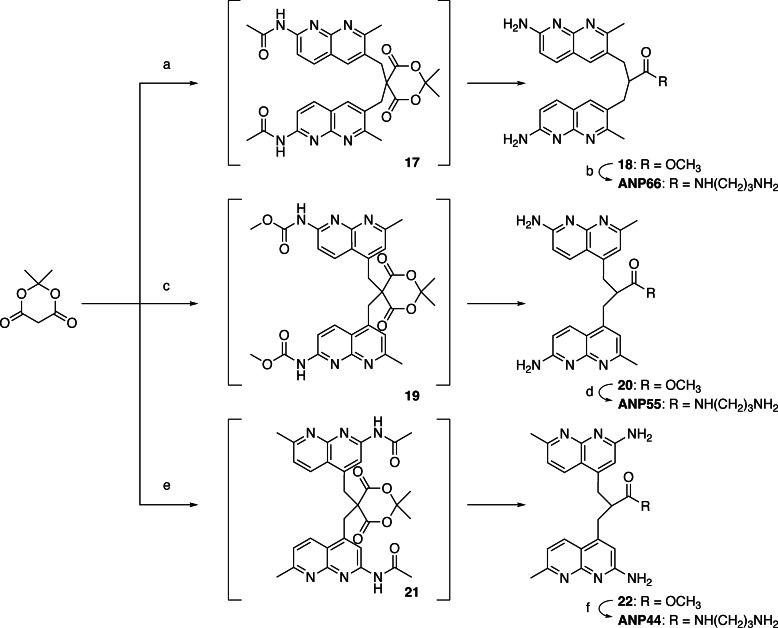
(a) **8**, K_2_CO_3_, DMF, r.t.,3 h; then, MeOH, K_2_CO_3_, reflux, overnight; (b) 1,3‐diaminopropane, 80 to 100 °C, overnight, 28 % (3 steps); (c) **10**, KI, K_2_CO_3_, DMF, r.t., overnight; then, MeOH, K_2_CO_3_, reflux, overnight, 74 % (2 steps); (d) 1,3‐diaminopropane, 100 °C, overnight, 29 %; (e) **16**, KI, K_2_CO_3_, DMF, r.t., overnight; then, MeOH, K_2_CO_3_, reflux, 6 h, 21 % (2 steps); (f) 1,3‐diaminopropane, 100 °C, overnight, 49 %.

**Scheme 5:**
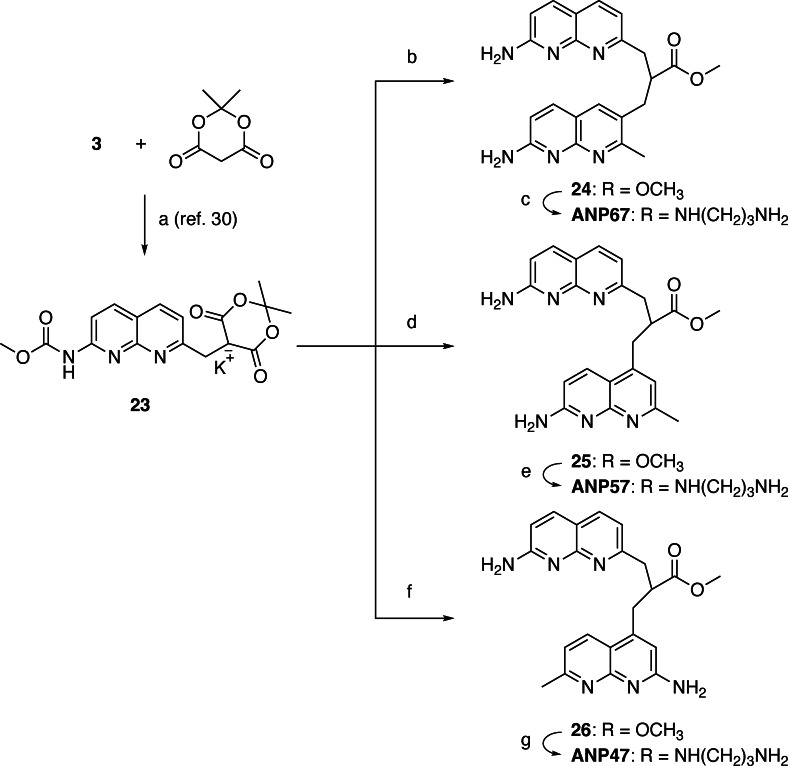
(a) K_2_CO_3_, KI, DMF, r.t., overnight, 68 %; (b) **8**, K_2_CO_3_, DMF, r.t., 4 h; then, MeOH, K_2_CO_3_, reflux, overnight, 47 % (2 steps); (c) 1,3‐diaminopropane, 100 °C, overnight, 32 %; (d) **10**, KI, K_2_CO_3_, DMF, r.t., overnight; then, MeOH, K_2_CO_3_, reflux, overnight, 40 % (2 steps); (e) 1,3‐diaminopropane, 100 °C, overnight, 30 %; (f) **16**, KI, K_2_CO_3_, DMF, r.t., overnight; then, MeOH, K_2_CO_3_, reflux, overnight; (g) 1,3‐diaminopropane, 100 °C, overnight, 9 % (3 steps).

**Scheme 6:**
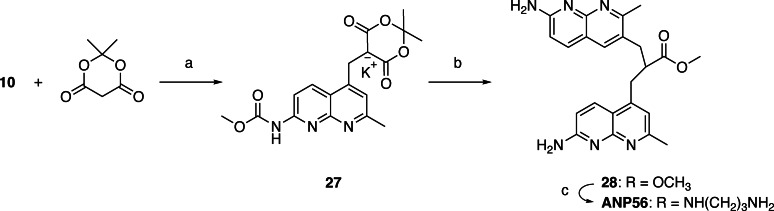
(a) K_2_CO_3_, KI, THF, r.t., 24 h, 58 %; (b) **8**, K_2_CO_3_, DMF, r.t., 22 h; then, MeOH, K_2_CO_3_, reflux, overnight; (c) 1,3‐diaminopropane, 100 °C, 18 h, 7 % (3 steps).

In the 2‐amino‐1,8‐naphthyridine, there are six positions that are potentially available for connecting with other heterocycles. It is well documented that a three‐sp3‐atom linker connecting two aromatic molecules facilitates the stacking.[^28^, ^29^, ^30^] To connect two 2‐amino‐1,8‐naphthyridine heterocycles, the position 2 is not suitable because of the presence of amino group at that position. In principle, two amino groups could be connected by one‐sp3‐atom linker, but the resulting aminal structure is not chemically stable. Connecting at the position 3 was also difficult because of the presence of the neighboring amino group. Thus, we designed ANPxy derivatives, where x and y denote the position of connections, by connecting at the position 4, 5, 6, and 7, and synthesized seven new molecules ANP44, ANP47, ANP55, ANP56, ANP57, ANP66, ANP67 in addition to the original ANP77. (Figure 2) Three homo‐dimers ANP66, ANP55, and ANP44 were connected at the same position for both naphthyridines, whereas four hetero dimers were connected at the different sites designated in their names. The hetero‐dimers contained the asymmetric center at the carbon bearing the amide side chain and are racemic mixtures. ANPxys except those connecting at the C7 position, have a methyl group at the C7 position due to the synthetic considerations. Therefore, molecular weight of ANPxys including ANP77 are varied from 430 to 458. Synthetic strategy of ANPxys is straight forward using the corresponding monomer‐halides and their coupling with Meldrum's acid[^31^, ^32^] followed by amide formation with 1,3‐diaminopropane. The synthesis of monomer unit **3** necessary for the preparation of ANPxy derivatives connected at C7 was reported in our previous paper,^[33]^ whereas the monomer unit **8** for 6‐substituted ANPxys was synthesized from 2‐amino‐7‐methyl‐1,8‐naphthyridine (**1**) by bromination at C6, lithiation followed by formylation, and transformation to iodide **8** in two steps (Scheme 1). The monomer unit **10** for 5‐substituted ANPxys was synthesized from 2,6‐diaminopyridine by coupling with 1‐chloro‐2,4‐pendandione and subsequent protection of the amino group as methyl carbamate (Scheme 2). The monomer unit **16** for 4‐substituted ANPxys was synthesized from 2,6‐dichloroisonicotic acid by ammonolysis to 2,6‐diaminoisonicotinic acid, followed by reduction of carboxylic acid to a primary alcohol to give 2,6‐diamino‐4‐hydroxymethlypyridine **13**. Coupling of **13** with 4,4‐dimethoxybutan‐2‐one produced 2‐amino‐1,8‐naphthyridine derivative **14** possessing hydroxymethyl substituent at the C4 position. The amino group was protected as an acetoamide, and the alcohol moiety was transformed into chloride to furnish the synthesis of **16**. (Scheme 3) Three homo‐dimers **ANP66**, **ANP55**, and **ANP44** were synthesized by coupling of Meldrum's acid with the monomer unit **8**, **10**, and **16**, respectively, followed by methanolysis and amidation with 1,3‐diaminopropane to provide the target molecules. (Scheme 4) Three hetero‐dimers **ANP67**, **ANP57**, and **ANP47** sharing the 7‐substituted naphthyridine were obtained by a reaction of potassium salt **23**, a mono coupling intermediate of Meldrum's acid, and **3** with the monomer unit **8**, **10**, and **16** followed by methanolysis and amidation, respectively. (Scheme 5) The last hetero‐dimer **ANP56** was obtained by a stepwise coupling of Meldrum's acid with **10** to give potassium salt **27**, and then with **8** followed by methanolysis and amidation. (Scheme 6)

### Absorption and Fluorescence Properties of ANPxys

**Figure 3:**
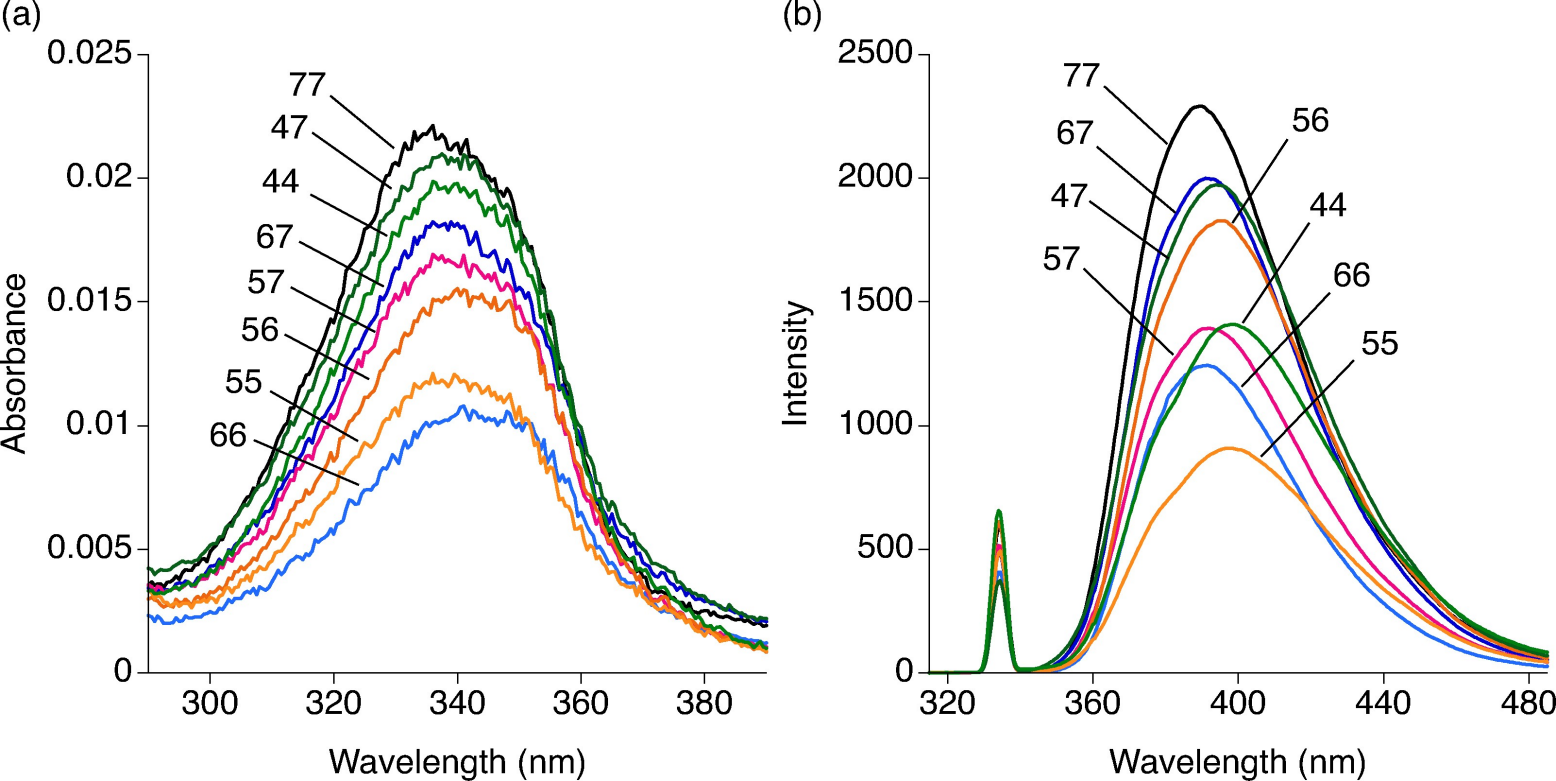
(a) Absorption spectra of ANPxy (1 μM) was measured in sodium cacodylate buffer (pH 7) and NaCl (100 mM) at room temperature. (b) Emission spectra was measured with the same solution for absorption measurements by exciting at 334 nm with a slit width of 2.5 nm and a response time of 0.5 s. Spectra correspond to ANPxy leveled with number.

Absorption and fluorescence spectra of ANPxys were measured in a buffer solution at pH 7. The absorption spectra showed a broad peak for each molecule and the maximum absorption wavelength (A_max_) ranged from 336 to 341 nm (Figure 3a and Table 1). All ANPxys exhibited fluorescence when excited at 334 nm and the emission maximum (E_max_) ranged from 389.5 to 398.5 nm. (Figure 3b) These results of spectra measurements showed that ANPxys possessed distinct electronic states although the core chromophore is almost the same. These differences are likely due to the combined effects of the position of substitution, the presence of methyl groups, and the degree of stacking of two naphthyridines. There was no apparent relationship between the number of methyl groups or the substitution patterns and A_max_ or E_max_. To estimate the degree of stacking of two chromophores, conformational search of ANPxys was performed. Based on the *pKa* of 6.3 for the 2‐amino‐7‐methyl‐1,8‐naphthyridine,^[27]^ mono‐protonated ANPxy was used for the simulation. For hetero‐dimeric ANPxys, two patterns of mono‐protonation were examined. The site of protonation was N1 because the N1 protonation was energetically more favorable than N8 protonation.^[34]^ For each mono‐protonated ANPxy, a set of 10,000 conformations was generated. These conformations were energy minimized using the OPLS4 force field^[35]^ with a continuous water model (Schrödinger, Maestro version 13.6). Among energy minimized structures, those within 8.36 KJ/mol (2 Kcal/mol) from the global energy minimum were further discussed. The probability of existence (*P*_exist_) of each conformer was calculated from the difference of the potential energy from that of global energy minimum. The probability of existence of stacked conformers (*P*_stack_) was calculated as the sum of *P*_exist_ of stacked conformers. (Table 2) ANP47 protonated at the 7‐substituted naphthyridine and ANP67 showed *P*_stack_ above 30 %, but *P*_stack_ of other ANPxys was not significant. These results indicate that the stacking of two heterocycles in ANPxys has, at most, a minor impact on the properties of these molecules.

**Table 1:** Absorption and Emission maximum of ANPxys.

	ANP44	ANP47	ANP55	ANP56	ANP57	ANP66	ANP67	ANP77
A_max_ (nm)	336.0	337.5	336.0	340.0	336.0	341.0	336.0	336.0
E_max_ (nm)	398.5	394.0	397.5	394.5	392.0	391.5	391.0	389.5
#	2	1	2	2	1	2	1	0

#: number of methyl group present within ANPxy.

**Table 2:** Existence probability of stacked conformation of mono‐protonated ANPxys.

	ANP44	ANP47	ANP55	ANP56	ANP57	ANP66	ANP67	ANP77
All conf^a)^	14	16 (14)	21	22 (17)	21 (21)	21	17 (22)	27
St conf^b)^	2	3 (4)	4	5 (4)	4 (4)	4	4 (4)	8
*P*_exist_ (%)	9	17 (39)	26	17 (18)	18 (24)	14	31 (34)	23

^a)^ The number of conformers within 8.36 KJ/mol (2 Kcal/mol) from the global minimum. The number in the parenthesis is for the mono‐protonated ANPxy at the N1 of y‐substituted 1,8‐naphthyridine. ^b)^ The number of stacked conformers.

### Effect of ANPxys on the Stabilization of DNA Duplexes

The effect of ANPxy binding on the stabilization of DNA duplexes (5 μM) containing C‐rich GTG/CCCC, GCG/CCCC, and GGCCCC/GGCCCC motifs were investigated by measuring UV‐melting temperature (*T*_m_) of duplexes 5’‐d(GTCCA GTG CAACG)‐3’/5’‐d(CGTTG CCCC TGGAC)‐3’, 5’‐d(GTCCA GCG CAACG)‐3’/5’‐d(CGTTG CCCC TGGAC)‐3’, and 5’‐d(GCAT GGCCCC TACG)‐3’/5’‐d(CGTA GGCCCC ATGC)‐3’ in 10mM sodium cacodylate buffer (pH 7.0) containing 100mM NaCl in the presence of 25 μM of ANPxy. (Tables 3, 4, 5) All ANPxys showed the increase of melting temperature (▵*T*_m_) of three DNAs with a varied magnitude and ANP77 recorded the highest ▵*T*_m_. Molecules ranked in the top three are ANP77, ANP56, ANP67 for GTG/CCCC DNA, ANP77, ANP47, ANP44 for GCG/CCCC DNA, and ANP77, ANP44, ANP47 for G2 C4/G2 C4 DNA. ANP57 was always ranked in the lowest group. These results showed that ANPxys have different binding and stabilizing effect for three DNA sequences although these molecules share the same heteroaromatic rings, indicating that the position of connection of two 2‐amino‐1,8‐naphthyridines significantly affected the binding.

**Table 3:** The *T*_m_ increase of GTG/CCCC DNA upon addition of ANPxy.

	Raw *T*_m_ data (°C)			*T*_m_ (°C)^a^	*ΔT*_m_ (°C)^b^	S.D.	remark
ANP77	–	–	–	46.1	16.5	0.8	Ref. [33]
ANP67	40.0	39.0	42.6	40.5	9.5	1.9	
ANP66	38.3	40.5	39.8	39.6	8.5	1.1	
ANP57	37.9	37.8	37.2	37.6	6.6	0.3	
ANP56	39.8	40.7	42.3	41.1	10.1	1.6	
ANP55	38.3	38.2	38.2	38.2	7.2	0.2	
ANP47	38.4	–	–	38.4	8.8	n.d.^c^	
ANP44	38.7	–	–	38.7	9.1	n.d ^c^	

a) average of *T*_m_s if multiple measurements are performed, b) increased *T*_m_ upon ANPxy addition. The *T*_m_ of DNA was 31.06 °C. c) S.D. was not determined due to a single measurement.

**Table 4:** The *T*_m_ increase of GCG/CCCC DNA upon addition of ANPxy.

	Raw *T*_m_ data (°C)			*T*_m_ (°C)^a^	*ΔT*_m_ (°C)^b^	S.D.	remark
ANP77	–	–	–	51.7	11.6	0.1	Ref. [33]
ANP67	46.3	45.3	47.0	46.2	6.1	0.8	
ANP66	43.8	45.2	45.9	45.0	4.9	1.0	
ANP57	46.2	45.3	44.7	45.4	5.3	0.8	
ANP56	46.6	46.6	46.0	46.4	6.3	0.3	
ANP55	45.2	45.4	44.7	45.1	5.0	0.3	
ANP47	48.6	–	–	48.6	8.5	n.d.^c^	
ANP44	47.5	–	–	47.5	7.4	n.d ^c^	

a) average of *T*_m_s if multiple measurements are performed, b) increased *T*_m_ upon ANPxy addition. The *T*_m_ of DNA was 40.10 °C. c) S.D. was not determined due to a single measurement.

**Table 5:** The *T*_m_ increase of G_2_C_4_/G_2_C_4_ DNA upon addition of ANPxy.

	Raw *T*_m_ data (°C)			*T*_m_ (°C)^a^	*ΔT*_m_ (°C)^b^	S.D.	remark
ANP77	52.2	51.9	–	52.1	20.0	–	
ANP67	43.4	43.1	44.4	43.6	11.5	0.7	
ANP66	42.5	44.1	45.0	43.9	11.8	0.7	
ANP57	41.2	40.0	41.4	40.9	8.8	0.8	
ANP56	44.4	44.5	45.8	44.9	12.8	0.8	
ANP55	44.3	44.5	44.1	44.3	12.2	0.2	
ANP47	47.0	–	–	47.0	14.9	n.d.^c^	
ANP44	47.9	–	–	47.9	15.8	n.d ^c^	

a) average of *T*_m_s if multiple measurements are performed, b) increased *T*_m_ upon ANPxy addition. The *T*_m_ of DNA was 32.10 °C. c) S.D. was not determined due to a single measurement.

### Interaction of ANPxys to RNAs Containing U/CC Motif

**Figure 4:**
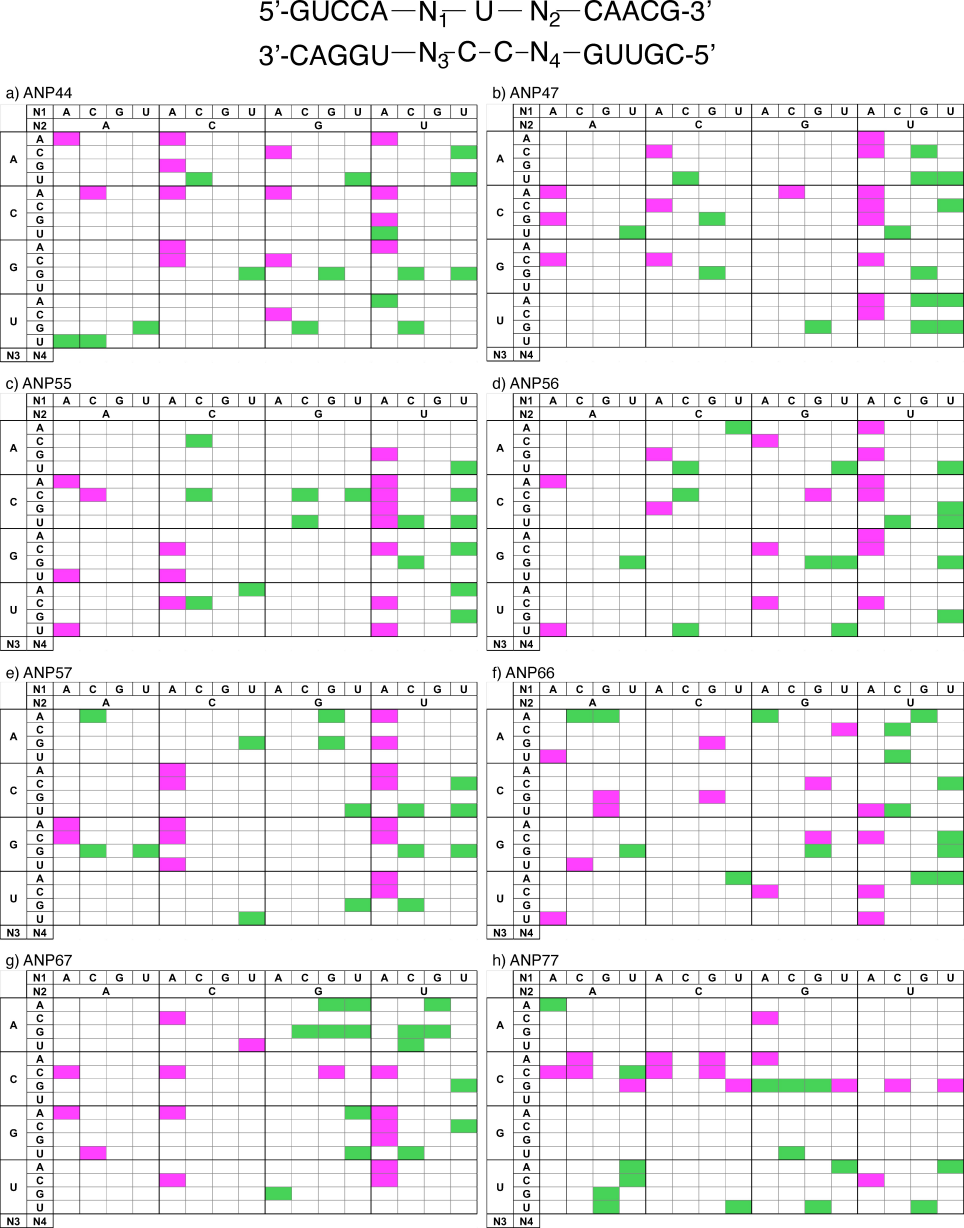
Heat maps of fluorescence changes of ANPxys in the presence of 256 RNAs having 5’‐N_1_UN_2_‐3’/3’‐N_3_CCN_4_‐5’ motifs. A) ANP44, b) ANP47, c) ANP55, d) ANP56, e) ANP57, f) ANP66, g) ANP67, and h) ANP77. Sequences showing top (magenta) and bottom (green) 15 F_c_ were shown. The number of F_c_ can be found in the Supporting (Figure S1).

*T*_m_ analysis of ANPxys showed that all ANPxys exhibited the ability of binding to three DNA motifs of GTG/CCCC, GCG/CCCC, and G2 C4/G2 C4 and stabilized it. To gain further insight into the ability of interaction of ANPxys by alternating the connection position of two naphthyridines, the comprehensive information for the interaction to the U/CC motif flanked by all possible combinations of the base pairs in the dsRNA were investigated. Thus, two RNA strands containing 5’‐N_1_UN_2_‐3’ and 5’‐N_4_CCN_3_‐3’ sequence, where N represents one of four nucleotide bases A, C, G and U, were hybridized to produce 256 kinds of RNA duplexes having the N_1_UN_2_/ N_4_CCN_3_ motif in the middle of the duplex. The binding of ANPxys to these RNAs were assessed by the degree of fluorescence changes, as we observed the quenching of ANP77 fluorescence upon binding to these RNAs.^[26]^ Fluorescence intensities of ANPxy (1 μM) were measured in the absence and presence of RNA duplex (1 μM) by exciting at 355 nm. The change in the fluorescence intensity (F_c_) of ANPxy with the RNA was calculated by dividing the fluorescence intensity with the highest fluorescence (F_max_) observed among 256 RNAs. (Figure S1) For all combinations of ANPxys and 256 RNAs, we observed the decrease in the fluorescence intensity to give F_c_ below 1 in the presence of RNA duplexes. Top and bottom 15 sequences in terms of F_c_ were shown in the matrix. (Figure 4) Each ANPxy showed a unique pattern of Fc for top (magenta) and bottom (green) sequences. Following characteristics for top 15 F_c_ patterns were observed; a) all compounds except ANP77 showed preference of the AUU/N_4_CCN_3_ (N_1_=A, N_2_=U) sequences, but preference of N_3_ and N_4_ depends on the molecule, b) AUC/N_4_CCN_3_ (N_1_=A, N_2_=C) are the second preference except ANP66, and the preferred nucleotides at N_3_ and N_4_ varied depending on the molecule, c) AUG/N_4_CCN_3_ (N_1_=A, N_2_=G) sequences were weakly preferred for ANP44, ANP56, ANP77, but not at all for ANP47, ANP55, ANP57, and ANP67, d) ANP77 is unique showing preference of cytosine at N_3_, and e) ANP57 showed preference of cytosine and guanine at N_3_. Regarding the low F_c_, each molecule showed a unique pattern with a weak preference of U at the N_1_ position.

**Figure 5:**
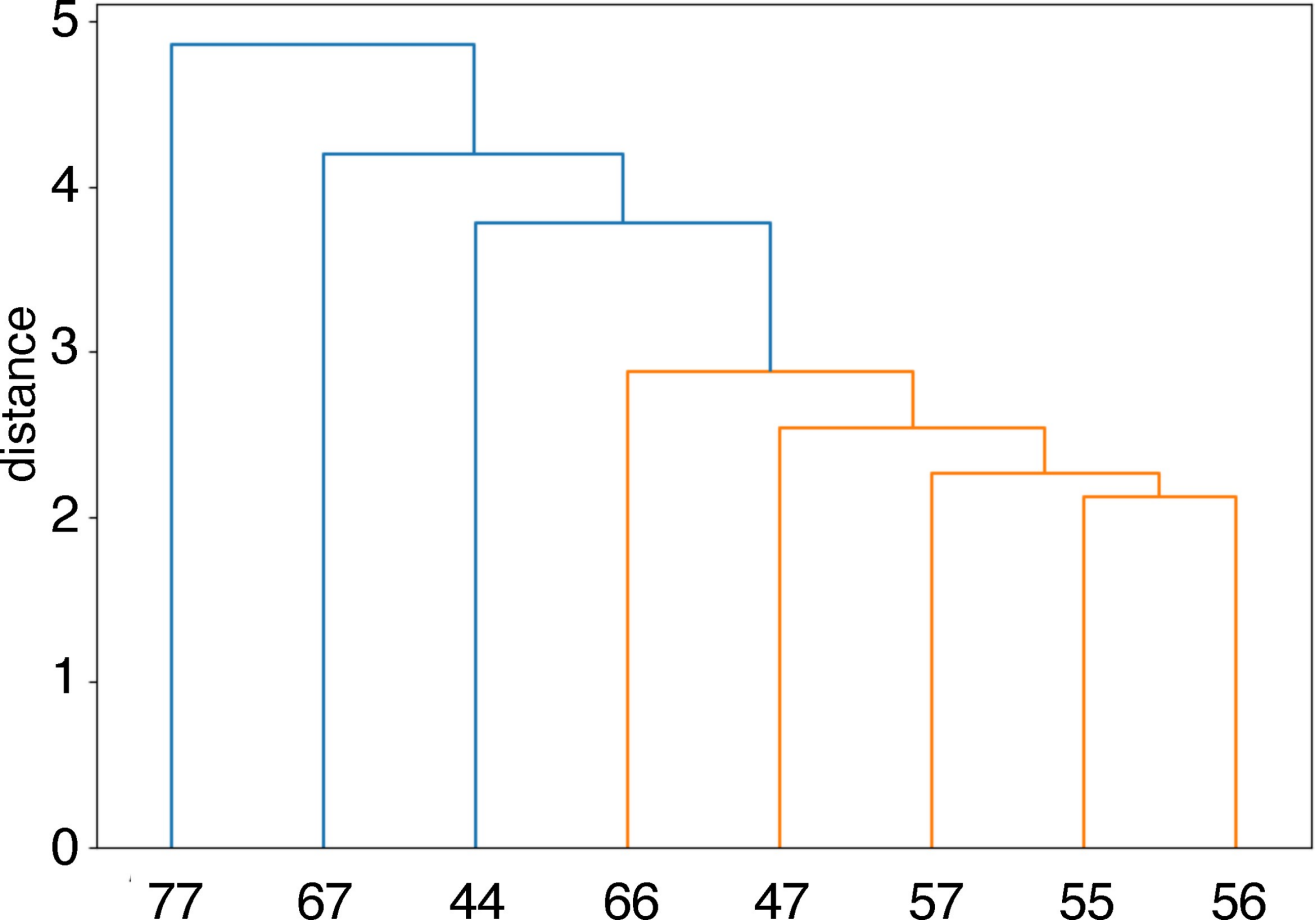
Hierarchical clustering of ANPxys by fluorescence change data with all 256 RNA sequences.

To further gain insight into the relationship between the structure and fluorescence changes, hierarchical clustering of 8 ANPxy molecules were carried out by using fluorescence changes with all 256 RNA sequences. The results of hierarchical clustering were represented by a dendrogram showing the distance between clusters. (Figure 5) The 8 ANPxys were grouped into two clusters, one group consisted of ANP77, ANP67, and ANP44, and the other consisted of ANP66, ANP47, ANP57, ANP55, and ANP56. The dendrogram showed that ANP55, ANP56, and ANP57 were close in the distance, suggesting the possibility that the connecting position at C5 of naphthyridine significantly affected the F_c_ patterns of these three molecules.

## Discussion

In our previous reports regarding the binding of ANP77 toward C/CC and T/CC motifs in dsDNA, ANP77 was revealed to be a high affinity binder to these unpaired DNA regions. ^[27]^ NMR structural analysis of ANP77 bound to the T/CC motif suggested that two naphthyridine heterocycles were placed directly opposite the two cytosines, with their hydrogen‐bonding surfaces oriented on the same side. While the precise structure of the complex could not be elucidated due to the presence of two diastereomers regarding the prochiral center of ANP77, the orientations and conformations of two naphthyridine were suggested to have significant impact on the binding of ANPxy derivatives. To address the relationship between the molecular structures having the same heterocycles and the binding properties, we have designed and synthesized seven new molecules, three homodimers ANP66, ANP55, and ANP44 and four heterodimers ANP67, ANP57, ANP56, and ANP47.

The absorption and emission spectra of eight ANPxy molecules showed that all molecules exhibited A_max_ in the range between 336 and 341 nm, and E_max_ in the range between 389.5 ad 398.5 nm with the intensity varied by the molecule. These emission spectra indicated that the emission is from the excited state of unstacked naphthyridines because the stacked naphthyridine produced an excimer like emission about 430 nm.^[33]^ Conformational analysis of eight molecules showed that the probability of stacked conformations was 39 % at the highest for the mono‐protonated ANP47. These spectral and conformational analysis showed that all ANPxys we discussed here took mainly unstacked conformation in the aqueous solution.

**Figure 6:**
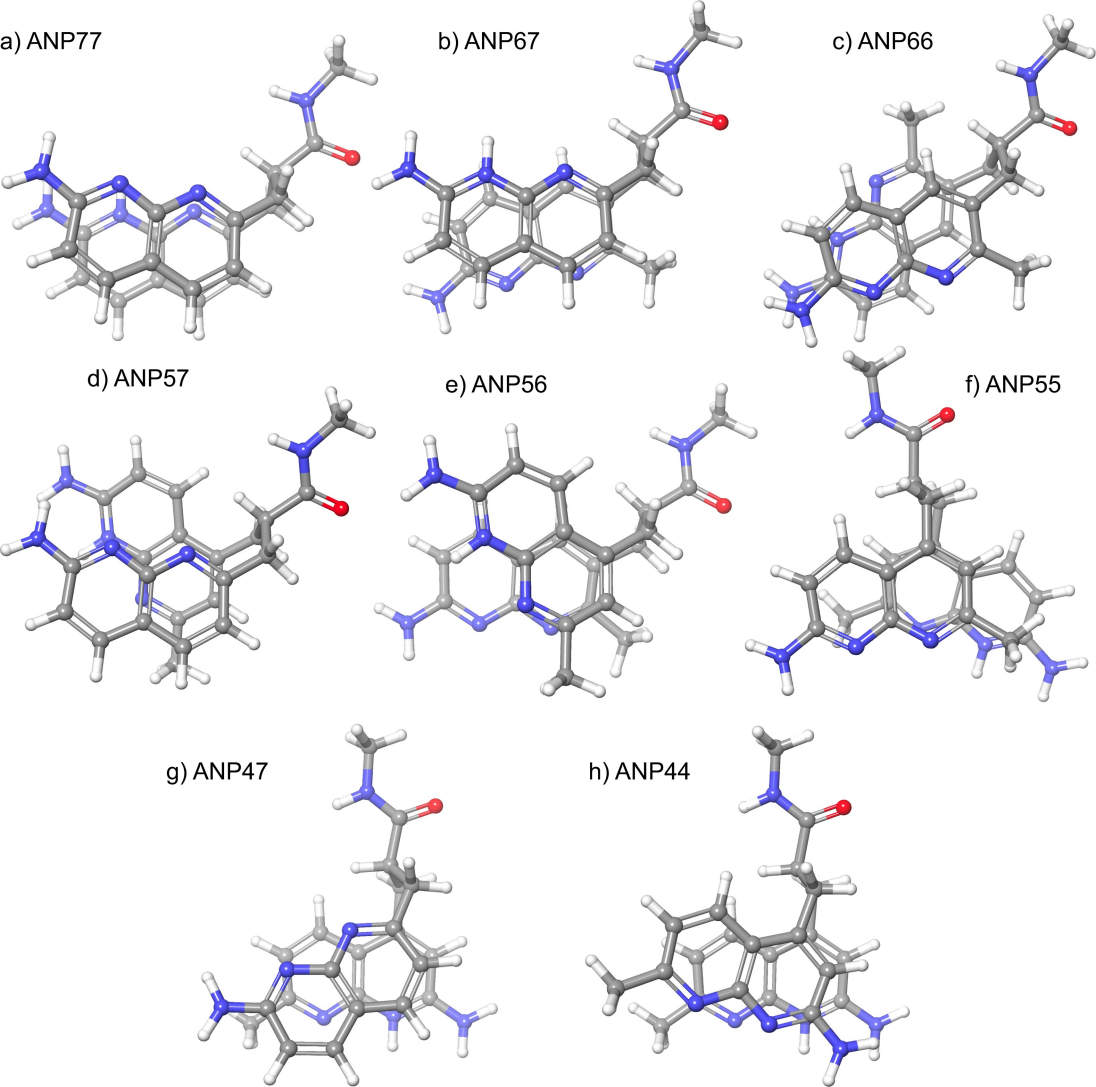
The lowest energy conformation of mono‐protonated ANPxy at N1 with a stacked structure among conformers obtained through conformational analysis. The detailed method for this analysis is described in the experimental section. The number in parentheses denotes the energy difference (KJ/mol) from the lowest‐energy conformer. For heterodimers, the protonated naphthyridine is indicated by “at x (or y).” The full list of conformational energies and the classification of “stacked” or “unstacked” can be found in the supporting material. A) ANP77 (+2.27), b) ANP67 protonated at 7 (0.00), c) ANP66 (+3.09), d) ANP57 protonated at 5 (+1.13), e) ANP56 protonated at 5 (+2.29), f) ANP55 (+1.70), g) ANP47 protonated at 4 (+1.05), and h) ANP44 (+4.05).

While ANP77 took unstacked conformation in the aqueous solution, we demonstrated that ANP77 bound to the T/CC motif in dsDNA with the stacked conformation. This is because the stacked conformation of ANP77 had hydrogen bonding surfaces fully complementary to the contiguous cytosines. To know the effect of connecting position on the structure of the stacked conformation, the lowest energy stacked conformation of eight ANPxy molecules obtained by conformational analysis was shown in Figure 6. Quite interestingly, ANP77 and ANP44 hold a similar stacked conformation showing the parallel stacked naphthyridines with the hydrogen bonding surfaces oriented on the same side, whereas ANP67 showed a parallel stacked conformation with the hydrogen bonding surfaces oriented on the opposite side. ANP55 hold an anti‐parallel stacked conformation with the hydrogen bonding surfaces oriented on the same side. Other four molecules ANP66, ANP57, ANP56, and ANP47 showed twisted conformations of two naphthyridines, and, therefore, the area of stacking of two heterocycles is much smaller than in parallel stacked conformations.

**Figure 7:**
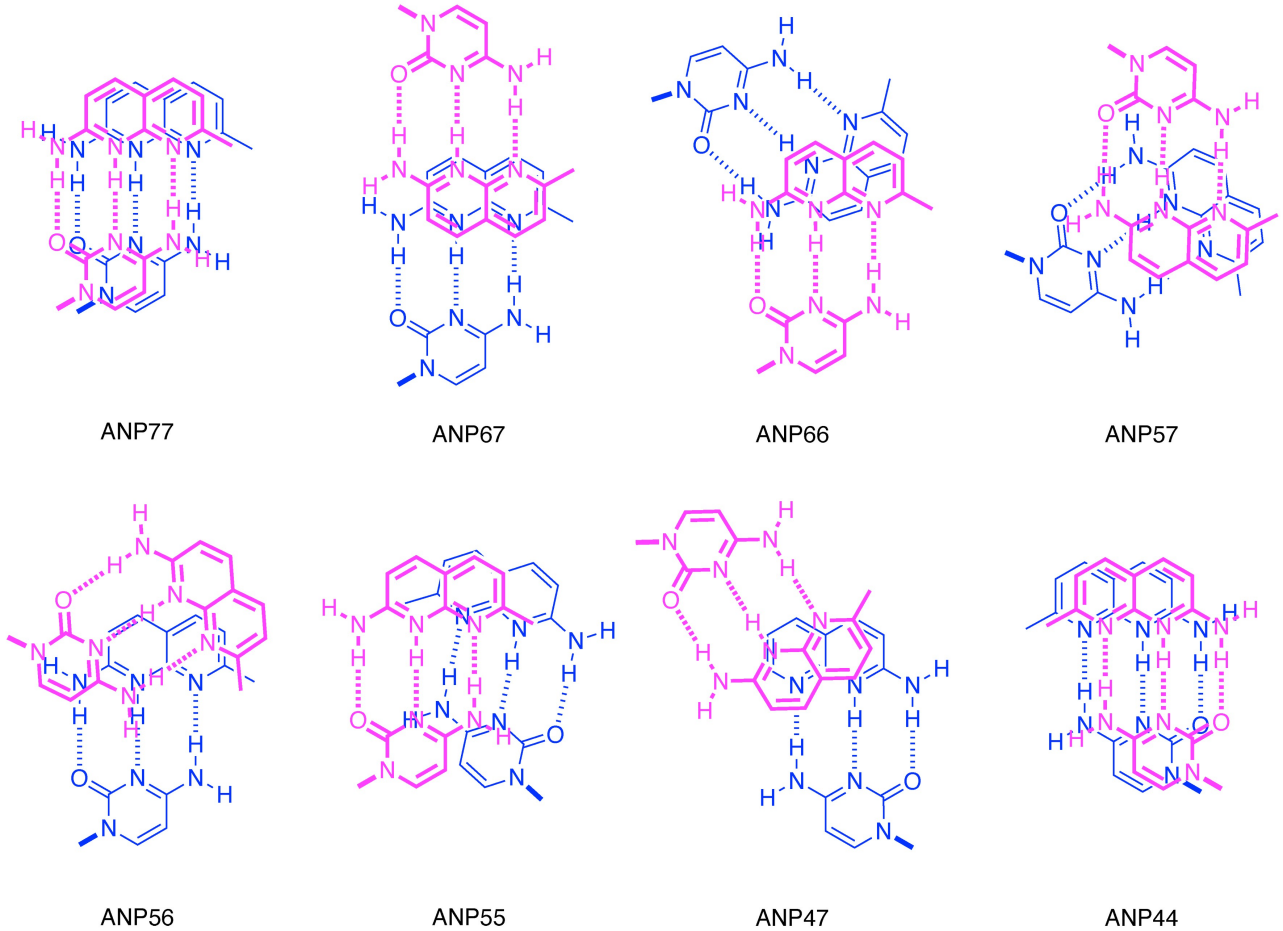
Illustrations of possible hydrogen‐bonding schemes of eight ANPxys with two cytosines. Two stacked 2‐amino‐1,8‐naphthyridine heterocycles in the lowest energy stacked conformation of the eight ANPxys (Figure 6) were hydrogen bonded to two cytosines. Both naphthyridines were protonated at N1. The “+” sings indicating a positive charge were omitted for clarity. The upper layer of the hydrogen‐bonded pair (shown in magenta) is depicted with a bold line over the lower layer of the hydrogen‐bonded pair (shown in blue). The glycosidic bond is also shown in bold.

*T*_m_ analysis of three DNA motifs showed that all eight ANPxys can bind to and stabilize the duplex with the degree of stability varying depending on the molecule. Despite of the differences in their available conformations, these observations that all eight ANPxys bound to these three DNA motifs are likely due to the structural flexibility of DNA motifs. Thus, the binding of ANPxys to the three DNA motifs could be rationalized by the induced‐fit and conformational selection models but not by the key‐and‐hole model.[^36^, ^37^] The conformational freedom of both the DNA motifs and the molecule may allow them to adopt a suitable orientation and structure for binding. Possible hydrogen‐bonding schemes of ANPxys with two cytosines are illustrated in Figure 7, based on the lowest‐energy conformation of ANPxy shown in Figure 6. As we speculated on induced‐fit and/or conformational selection models for binding, these hydrogen‐bonding schemes are provided merely to expand the conceptual understanding of how these eight ANPxy compounds interact with cytosine‐rich DNA motifs. In each illustration where ANPxy binds to two cytosines, the position and angle between the two glycosidic bonds connecting cytosine and deoxyribose (shown in bold) differ significantly. ANP77 and ANP44, which were classified into the same group by hierarchical clustering based on fluorescence quenching efficiency with U/CC RNAs, show that the two glycosidic bonds are oriented almost in the same direction. ANP67, the third molecule in this cluster, exhibits an angle between the two glycosidic bonds similar to that found in B‐form DNA. In contrast, the molecules in the second cluster exhibit glycosidic bond angles that differ from those observed in the first cluster. Considering that all eight compounds bind to the three types of cytosine‐rich DNA motifs, albeit with different binding affinities, the binding of compounds where two heterocycles are connected by a short linker and are expected to stack favorably in water would likely require a significant structural change in the nucleic acid to facilitate hydrogen bond formation between the hydrogen‐bonding groups at the edges of the heterocycles and the bases on the nucleic acid. Further discussions regarding the mode of the binding of ANPxys to these DNA motifs awaits the structural analysis of ANPxy‐bound DNA complexes.

Having confirmed the binding of ANPxys to three DNA motifs, we then focused on the interaction of ANPxys to RNA motifs involving 5’‐N_1_UN_2_‐3’/5’‐N_4_CCN_3_‐3’ sequence motifs. There are 256 kinds of N_1_UN_2_/N_4_CCN_3_ motifs, which could be prepared by hybridization of 16 N_1_UN_2_ RNA strands with 16 N_4_CCN_3_ RNAs. The binding of each ANPxy to these 256 RNAs were assessed by the changes of ANPxy fluorescence in the absence and presence of RNAs. As shown in Figure S1, eight molecules showed distinct patterns of fluorescence quenching depending on the nucleotides locating N_1_, N_2_, N_3_, and N_4_ positions. These nucleotides flanking the U/CC motif, in principle, can provide a unique structural environment for the ANPxy binding. The top and bottom 15 sequences in terms of the fluorescence changes shown in Figure 4 highlight the characteristics of each molecule. Hierarchical clustering of 8 ANPxys resulted in two clusters; one consisted of ANP77, ANP67, and ANP44, and the other consisted of ANP66, ANP47, ANP57, ANP55, and ANP56. The ANPxy molecules in the second cluster showed significant fluorescent changes in the RNA sequence involving A in N_1_. ANPxy molecules in two clusters are coincidentally classified by the degree of stacking of the lowest stacked conformations. The molecules in the first cluster ANP77, ANP67, and ANP44 had a parallel stacked conformation, whereas other 5 molecules in the second cluster had a twisted stacked conformation. It is evident that the connecting two naphthyridines at different positions provided a variety of molecules having the same hydrogen bonding surfaces, and these molecules showed characteristic properties on the binding DNA motifs and the interaction to the RNA motifs. The conformational differences are likely one of contributes for these properties.

We synthesized 8 ANPxy molecules containing two 2‐amino‐1,8‐naphthyridine heterocycles and discussed the fluorescence and binding properties with their available conformations in unbound state. ANP77 representing the ANPxys is one of the mismatch binding ligands with the high affinity to specific DNA motifs. Our studies on the mismatch binding ligands consisted of two heterocycles demonstrated the high potential of these class of molecules for the binding to the mismatched base pairs in DNAs and RNAs. We believe these studies described here provided with the basic data regarding the small molecule interaction to RNA motifs of ANPxys and may be extended to the design of small molecules targeting RNAs having multiple heterocycles with various conformations.

## Experimental Section

### General

Reagents and solvents were purchased from standard suppliers and used without further purification. Reactions were monitored by TLC on plates precoated with Merck silica gel 60 F254 or Wako NH_2_ silica Gel 60 F254. Spots were visualized with UV light, ninhydrin or iodine. Wakogel C‐200 or Fuji Silysia Chemical Chromatorex NH‐DM1020 were used for flash chromatography on silica gel. ^1^H and ^13^C NMR spectra were measured on JEOL JNM‐LA400 or LA600 spectrometers. The chemical shifts (*δ*) are expressed in ppm relative to residual solvent as an internal standard and coupling constants (*J* values) are given in Hz. The multiplicity was expressed as follows: s=singlet, d=doublet, t=triplet, q=quartet, m=multiplet, br=broad. ESI‐TOF mass spectra were recorded on a JEOL AccuTOF JMS−T100 N mass spectrometer.

### UV and Fluorescence Measurements

Absorption spectra of ANPxy (1 μM) was measured in sodium cacodylate buffer (pH 7) and NaCl (100 mM) was measured at room temperature. Emission spectra was measured with the same solution for absorption measurements by exciting at 334 nm with a slit width of 2.5 nm and a response time of 0.5 s.

### Conformational Analysis

Conformational analysis was performed on the ANPxy derivative by replacing the amide moiety of ANPxy with *N*‐methyl amide and both naphthyridine nitrogen atoms at the N1 position were protonated. Five C−C single bonds and a C−N bond in the compound was systematic torsional sampling (SPMC) method implemented in Maestro Molecular modeling package. A detail setting is as follows: Force Field, OPLS4 with solvent water; Minimization, PRCG, maximum iterations of 5000, converge on Gradient, and convergence threshold of 0.05; Conformational search, SPMC, non‐automatic setup, torsion sampling with intermediate frequency, comparison atom is heavy atom only; Maximum number of steps, 20,000; Use 100 steps per rotatable bond; energy window for saving the structures was 50 KJ/mol. The conformations within 8.36 KJ/mol (2 Kcal/mol) from the global minimum were manually investigated if two naphthyridine heterocycles were stacked with each other, and the number of stacked and non‐stacked conformations were counted. The probability of existence (*P*_exist_) of stacked conformations was obtained as the sum of *P*_exist_ of each stacked conformer calculated from the energy difference from the global minimum with the equation below at 300 K:


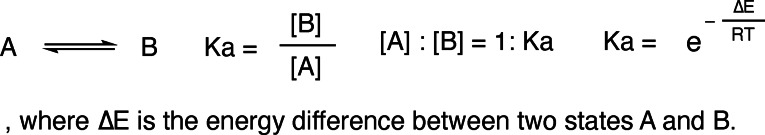


### UV‐thermal Denaturing Temperature Measurements

Thermal melting curves were measured for 5 μM DNA duplexes 5’‐GTCCA GTG CAACG‐3’/5’‐d(CGTTG CCCC TGGAC)‐3’, 5’‐GTCCA GCG CAACG‐3’/5’‐d(CGTTG CCCC TGGAC)‐3’ or 5’‐d(GCAT GGCCCC TACG)‐3’/5’‐d(CGTA GGCCCC ATGC)‐3’ in 10mM sodium cacodylate buffer (pH 7.0) containing 100mM NaCl with or without ANPxy (25 μM) by increasing the temperature from 2 °C to 90 °C with heating rate of 1 °C/min. The underlined sequence forms an internal loop with some unpaired bases. The data about ANP77 is cited from our previous research.^[27]^

### Fluorometric Screening

The fluorometric screening was performed with equimolar mixture (1 μM) of **ANPxy** and 256 kinds of dsRNA containing 5’‐N_1_UN_2_‐3’/3’‐N_3_CCN_4_‐5’ motifs in sodium cacodylate buffer (10 mM, pH 7) and sodium chloride (100 mM) using a 384‐well assay plate. The fluorescence of each well containing sample (15 μL volume) was measured using a microplate reader (Mithras LB 940) with 355 nm excitation and 410 nm emission filters at room temperature. Samples lacking the RNA duplex or both dsRNA and ANPxy was also dispensed to the plate and measured the fluorescence. All the RNAs used for the fluorometric screening were procured from Gene Design Inc. (Osaka, Japan). The change in the fluorescence intensity (F_c_) of ANPxy with the RNA was calculated by dividing the fluorescence intensity with the maximum fluorescence (F_max_) observed among 256 RNAs. The outliers of fluorescence intensity were not included for the determination of the F_max_.

### Synthesis of ANPxys

#### 2‐amino‐6‐bromo‐7‐methyl‐1,8‐naphthyridine (4)

Bromine (320 μL, 6.20 mmol) was added to 2‐amino‐7‐methyl‐1,8‐naphthyridine (1.0 g, 6.28 mmol) in 1,4‐dioxane/water (1/1) at 0 °C and stirred for 1 h. Then, the reaction mixture was stirred at room temperature for 3.5 h. The reaction mixture was extracted with chloroform and purified by column chromatography with chloroform to chloroform/methanol (98/2) as an eluent and dried in a high vacuum to yield **4** (0.99 g, 66 %). ^1^H NMR (400 MHz, CD_3_OD) *δ* 8.22 (s, 1H), 7.86 (d, *J*=8.7 Hz, 1H), 6.84 (d, *J*=8.7 Hz, 1H), 2.72 (s, 3H). HRMS (ESI+): *m/z* calcd. for [C_9_H_8_BrN_3_ + Na]^+^: 259.9800, found: 259.9791.

#### *N*‐(6‐(hydroxymethyl)‐7‐methyl‐1,8‐naphthyridin‐2‐yl)acetamide (7)

*n*‐Butyllithium solution 1.55 M in hexane (6.05 mL, 9.38 mmol) was added dropwise to a solution of **4** (680 mg, 2.86 mmol) in anhydrous THF (28 mL) at –100 °C under Ar and the mixture was stirred for 1 h. Then anhydrous DMF (1.33 mL, 17.16 mmol) in THF (2.0 mL) was added dropwise to the reaction mixture at –100 °C under Ar and stirred for 2 h. Then, the reaction mixture was allowed to warm to room temperature. The mixture was quenched by water, extracted with CHCl_3_, dried over MgSO_4_, and evaporated *in vacuo*. The crude product was roughly purified by column chromatography on silica gel (CHCl_3_ to CHCl_3_/MeOH=97 : 3) to give a crude 2‐amino‐6‐formyl‐7‐methyl‐1,8‐naphthyridine (**5**) containing some impurities. Aliquot of the sample was further purified to confirm the structure. ^1^H NMR (400 MHz, CDCl_3_) *δ* 10.32 (s, 1H), 8.38 (s, 1H), 7.94 (d, *J*=8.8 Hz, 1H), 6.78 (d, *J*=8.8 Hz, 1H), 5.25 (br, 2H), 3.00 (s, 3H). HRMS (ESI+): *m/z* calcd. for [C_10_H_9_N_3_O + Na]^+^: 210.0644, found: 210.0633.

To a solution of crude **5** (ca. 0.3 g) in Pyridine 6 mL was added acetic anhydride (2.0 mL) and the resulting solution was stirred for 2 h under reflux condition. The mixture was evaporated *in vacuo*, diluted with water, and extracted with chloroform. The organic phase was dried over MgSO_4_, and evaporated *in vacuo* to give crude *N*‐(6‐formyl‐7‐methyl‐1,8‐naphthyridin‐2‐yl)acetamide (**6**) with some impurities (377 mg) as a pale‐yellow solid. The major component was confirmed as **6** by NMR. This pale‐yellow solid was utilized for the next reaction without further purification. ^1^H NMR (600 MHz, CDCl_3_) δ 10.37 (s, 1H), 8.56 (s, 1H), 8.56 (d, *J*=8.9 Hz, 1H), 8.51‐8.43 (br, 1H), 8.28 (d, *J*=8.9 Hz, 1H), 3.06 (s, 3H), 2.30 (s, 3H).

To a solution of **6** containing impurities (ca. 0.38 g) in EtOH (20 mL), NaBH_4_ (65 mg, 1.72 mmol) was added at ice cool condition and the resulting mixture was stirred for 1 h at room temperature under Ar. The reaction was quenched by adding AcOH (0.40 mL) and the mixture was concentrated *in vacuo* with azeotropic removal of acetic acid with toluene. The crude product was purified by column chromatography on silica gel (CHCl_3_ to CHCl_3_/MeOH=98 : 2) to give **7** (229 mg, 35 %, 3 steps from **4**) as a brown solid. ^1^H NMR (400 MHz, CD_3_OD) δ 8.39 (d, *J*=9.2 Hz, 1H), 8.31 (d, *J*=8.8 Hz, 1H), 8.25 (s, 1H), 4.81 (s, 2H), 2.72 (s, 3H), 2.25 (s, 3H). ^13^C NMR (150 MHz, CD_3_OD) δ 172.5, 162.8, 155.1, 154.6, 140.1, 136.0, 134.8, 120.3, 115.8, 62.1, 24.3, 22.4. HRMS (ESI+): *m/z* calcd. for [C12H13 N3O2 + Na]^+^: 254.0906, found: 254.0895.

#### *N*‐(6‐(iodomethyl)‐7‐methyl‐1,8‐naphthyridin‐2‐yl)acetamide (8)

To a solution of **7** (194 mg, 0.84 mmol) and triethyl amine (0.5 mL) in anhydrous DCM (20 mL), methanesulfonyl chloride (194 μL, 2.51 mmol) was added at 0 °C and the resulting mixture was stirred for 3 h at room temperature under Ar. The mixture was diluted with water and extract with CHCl_3_. The organic phase was dried over MgSO_4_, and evaporated *in vacuo* to give mesylate derivative of **7**. Then the mesylate was dissolved in acetone (32 mL), NaI (420 mg, 2.80 mmol) was added to the mixture. The mixture was heated to reflux for 2 h under Ar. Evaporated the acetone, added water and extracted with CHCl_3_. The organic phase was dried over MgSO_4_, and evaporated *in vacuo* to give **8** (140 mg, 49 %) as a pale green solid. **8** was stored at −20 °C under Ar atmosphere because it was unstable in the air. ^1^H NMR (400 MHz, CDCl_3_) δ 8.46 (d, *J*=8.4 Hz, 1H), 8.32 (brs, 1H), 8.12 (d, *J*=9.2 Hz, 1H), 8.03 (s, 1H), 4.59 (s, 2H), 2.79 (s, 3H), 2.28 (s, 3H). ^13^C NMR (150 MHz, CDCl_3_) δ 169.4, 161.9, 153.9, 153.6, 138.8, 136.1, 131.1, 119.2, 114.8, 25.1, 23.3. HRMS (ESI+): *m/z* calcd. for [C_12_H_12_N_3_OI + Na]^+^: 363.9923, found: 363.9910.

#### 5‐(chloromethyl)‐7‐methyl‐1,8‐naphthyridin‐2‐amine (9)

To a solution of 2,6‐diaminopyridine (163 mg, 1.49 mmol) in phosphoric acid (2 mL, 85 % with water), 1‐chloropentane‐2,4‐pentandione (prepared according to the reported procedure)^[38]^ (260 mg, 1.93 mmol) was added and stirred at 80 °C for 20 h. The reaction mixture was neutralized with potassium carbonate solution and extracted with chloroform. The chloroform part was dried over MgSO_4_, and evaporated *in vacuo*. The resulting crude product was purified by column chromatography with chloroform to chloroform/methanol (97/3) as an eluent to give **9** (46.9 mg, 15 % yield). ^1^H NMR (600 MHz, CD_3_OH) δ 8.19 (d, *J*=9.0 Hz, 1H), 7.18 (s, 1H), 6.87 (d, *J*=9.0 Hz, 1H), 4.95 (s, 2H), 2.61 (s, 3H). ^13^C NMR (150 MHz, CD_3_OD) δ 161.2, 161.0, 156.4, 144.4, 133.7, 118.3, 112.9, 112.3, 40.8, 23.4. HRMS (ESI+): *m/z* calcd. for [C10H10 N3Cl + H]^+^: 208.0642, found: 208.0634.

#### Methyl (5‐(chloromethyl)‐7‐methyl‐1,8‐naphthyridin‐2‐yl)carbamate (10)

To a solution of **9** (160 mg, 0.77 mmol) in anhydrous chloroform (12 mL) cooled in an ice bath, methyl chloroformate (437 mg, 358 μL, 4.6 mmol) and potassium carbonate (640 mg, 4.6 mmol) were added and the mixture was stirred at room temperature for 61 h under Ar. The mixture was extracted with chloroform, dried over Na_2_SO_4_, and concentrated *in vacuo*. The resulting crude product was purified by column chromatography with ethyl acetate as an eluent and dried *in vacuo* to give **10** (191 mg, 93 % yield). ^1^H NMR (400 MHz, CDCl_3_) δ 8.40 (d, *J*=9.2 Hz, 1H), 8.33 (d, *J*=9.2 Hz, 1H), 7.72 (s, 1H), 7.30 (s, 1H), 4.90 (s, 2H), 3.85 (s, 3H), 2.75 (s, 3H). ^13^C NMR (100 MHz, CDCl_3_) δ 163.3, 155.2, 153.6, 153.3, 143.0, 134.9, 121.3, 116.0, 112.7, 52.8, 41.5, 25.6. HRMS (ESI+): *m/z* calcd. for [C_12_H_12_N_3_O_2_Cl + H]^+^: 266.0697, found: 266.0689.

#### (2,6‐diaminopyridin‐4‐yl)methanol (13)

The mixture of 2,6‐dichloroisonicotinic acid (3.49 g, 18.2 mmol), copper powder (347 mg, 5.50 mmol), and 28 % aqueous ammonia (31.2 mL) in a sealed tube was stirred at 180 °C for 20.5 h. After cooling to room temperature, copper was removed by filtration and 1 M HCl was added until the filtrate reached pH 5. The precipitate 2,6‐dichloroisonicotinic acid (**11**) was filtered off and dried *in vacuo*. To the resulting solid placed in an ice bath, 30.0 mL anhydrous ethanol and 30.5 mL 4 M HCl/ethyl acetate was added and the mixture was stirred at 80 °C for 18 h under Ar atmosphere. After concentrating the mixture, the residue was dissolved in water, and 28 % aqueous ammonia was added until the pH became 8. The produced solid was removed by filtration and the filtrate was extracted with ethyl acetate. The organic phase was dried with MgSO_4_ and concentrated *in vacuo*. The resulting crude product was purified by column chromatography on silica gel (using chloroform as an eluent) to afford Ethyl 2,6‐diaminoisonicotinate (**12**) as a yellow solid (1.25 g, 38 %). ^1^H NMR (600 MHz, DMSO‐*d*_6_) δ 6.07 (s, 2H), 5.66 (s, 4H), 4.19 (q, *J*=7.2 Hz, 2H), 1.23 (t, *J*=7.2 Hz, 3H).

Lithium aluminum hydride (625 mg, 16.5 mmol) was placed in a two‐neck flask and cooled in an ice bath. A THF solution (20 mL) of **12** (974 mg, 5.4 mmol) was slowly added dropwise at 0 °C under Ar atmosphere, then the resulting mixture was stirred at room temperature for 3 h. The reaction mixture was cooled on ice, and 0.62 mL of water, 0.62 mL of 10 % sodium hydroxide solution, and 2.0 mL of water were added dropwise and stirred for 1 h. The reaction mixture was filtered through celite and the solid was washed with a large amount of methanol. The solvent was removed under reduced pressure and the residue was purified by column chromatography on silica gel (using chloroform as an eluent) to afford **13** (647 mg, 86 %) as a yellow solid. ^1^H NMR (600 MHz, DMSO‐*d*_6_) δ 5.57 (s, 2H), 5.21 (s, 4 H), 4.95 (t, *J*=5.4 Hz, 1H), 4.16 (d, *J*=5.4 Hz, 2H). HRMS (ESI+): m/z cald. for [C_6_H_9_N_3_O + H]^+^: 140.0824, found: 140.0816.

#### (2‐amino‐7‐methyl‐1,8‐naphthyridin‐4‐yl)methanol (14)

**13** (305.0 mg, 2.19 mmol) was dissolved in phosphoric acid (3.0 mL, 85 % with water). To the solution, 4,4‐dimethoxybutan‐2‐one (378 μL, 2.85 mmol) was slowly added dropwise and the mixture was stirred at 90 °C for 15 h. After cooled down to r.t., the reaction mixture was neutralized with aqueous ammonia (28 %). The resulting salts were removed and washed with a large amount of methanol. The combined filtrate was evaporated *in vacuo* and the residue was purified by column chromatography on amino silica gel (using mixture of ethyl acetate and methanol as an eluent) to afford **14** (73.8 mg, 18 %) as a white solid. ^1^H NMR (600 MHz, CD_3_OD) δ 8.08 (d, *J*=8.4 Hz, 1H), 7.09 (d, *J*=7.2 Hz, 1H), 6.91 (s, 1H), 4.92 (s, 2H), 2.59 (s, 3H). HRMS (ESI+): m/z cald. for [C_10_H_11_N_3_O + Na]^+^: 212.0800, found: 212.0796.

#### *N*‐(4‐(hydroxymethyl)‐7‐methyl‐1,8‐naphthyridin‐2‐yl)acetamide (15)

A mixture of **14** (131.0 mg, 0.69 mmol) and acetic anhydride (5.4 mL) was stirred at 60 °C for 3.5 h. The excess acetic anhydride was removed under reduced pressure to afford crude **15** in addition to *N,O*‐bisacetylated compound. The crude **15** was dissolved in 5.4 mL methanol, and potassium carbonate (12.4) was added. The mixture was stirred at room temperature for 1.5 h. A saturated aqueous ammonium chloride solution was added to quench the reaction, and the solvent was removed under reduced pressure. The resulting crude purified by column chromatography on amino silica gel (using mixture of ethyl acetate and methanol as an eluent) to afford **15** (88.1 mg, 55 %) as an off‐white solid. ^1^H NMR (600 MHz, DMSO‐*d_6_*) δ 10.86 (s, 1H), 8.38 (s, 1H), 8.25 (d, *J*=8.4 Hz, 1H), 7.33 (d, *J*=7.2 Hz, 1H), 5.58 (t, *J*=5.4 Hz, 1H), 4.93 (d, *J*=4.8 Hz, 2H), 2.60 (s, 3H), 2.13 (s, 3H). HRMS (ESI+): m/z cald. for [C_12_H_13_N_3_O_2_ + Na]^+^: 254.0906, found: 254.0896.

#### *N*‐(4‐(chloromethyl)‐7‐methyl‐1,8‐naphthyridin‐2‐yl)acetamide (16)

**15** (80.0 mg, 0.35 mmol) was placed in a flask and cooled down to 0 °C in the ice bath. Then, 2.1 mL thionyl chloride was added slowly and stirred at room temperature for 2 h under Ar. The excess thionyl chloride was removed under reduced pressure and the resulting crude products were purified by column chromatography on amino silica gel (using mixture of chloroform and methanol as eluent) to afford **16** (40.9 mg, 47 %) as a yellow solid. ^1^H NMR (400 MHz, CDCl_3_) δ 8.52 (s, 1H), 8.46 (s, 1H), 8.29 (d, *J*=8.4 Hz, 1H), 7.34 (d, *J*=8.8 Hz, 1H), 4.92 (s, 2H), 2.76 (s, 3H), 2.27 (s, 3H). HRMS (ESI+): m/z cald. for [C_12_H_12_ClN_3_O + Na]^+^: 272.0567, found: 272.0556.

#### Methyl 3‐(7‐amino‐2‐methyl‐1,8‐naphthyridin‐3‐yl)‐2‐((7‐amino‐2‐methyl‐1,8‐naphthyridin‐3‐yl)methyl)propanoate (18)

A solution of **8** (80 mg, 0.23 mmol), potassium carbonate (36.2 mg, 0.26 mmol), and Meldrum's acid (16.7 mg, 0.12 mmol) in DMF (2 mL) was stirred at room temperature for 3 h under Ar. The mixture was evaporated *in vacuo*, and anhydrous methanol was added to the residue. The resulting solution was stirred under Ar at reflux for overnight. Methanol was evaporated *in vacuo* and the resulting crude material was purified by column chromatography on silica gel (using chloroform/methanol, 97/3 to 70/30 as an eluent) to afford **18** with inorganic impurities as a white solid. Aliquot of crude material was purified to get pure **18** to confirm the structure. ^1^H NMR (600 MHz, DMSO‐*d*_6_) δ 7.91 (d, *J*= 9.0 Hz, 2H), 7.86 (s, 2H), 7.01 (brs, 4H), 6.78 (d, *J*=9.0 Hz, 2H), 3.37 (s, 3H), 3.02–2.96 (m, 5H), 2.53 (s, 6H). ^13^C NMR (150 MHz, DMSO‐*d*_6_) δ 174.4, 160.3, 159.1, 154.6, 137.2, 136.5, 126.3, 114.9, 112.6, 51.4, 46.4, 34.3, 22.7. HRMS (ESI+): *m/z* calcd. for [C23H24 N6O2 + 2H]^2+^: 209.1059, found: 209.1051.

#### 3‐(7‐amino‐2‐methyl‐1,8‐naphthyridin‐3‐yl)‐2‐((7‐amino‐2‐methyl‐1,8‐naphthyridin‐3‐yl)methyl)‐*N*‐(3‐aminopropyl)propanamide (ANP66)

A solution of crude **18** in 1,3‐diaminopropane (2 mL) was stirred at 100 °C overnight. After the mixture was concentrated *in vacuo*, the residue was diluted with a small amount of water. The resulting crude product was purified by HPLC by using 0–30 % water‐acetonitrile (0.1 % AcOH) to afford **ANP66** (15.5 mg, 28 % for 3 steps from **8**). 1H NMR (600 MHz, CD_3_OD) δ 7.85 (d, *J*=8.4 Hz, 2H), 7.80 (s, 2H), 6.81 (d, *J*=9.0 Hz, 2H), 3.13 (dd, *J*=14.0, 10.2 Hz, 2H), 3.03 (dd, *J*=14.1, 4.8 Hz, 2H), 2.90‐2.87 (m, 3H), 2.65 (s, 6H), 2.37 (t, *J*=7.2 Hz, 2H), 1.36‐1.31 (m, 2H); ^13^C NMR (150 MHz, CD_3_OD) δ 176.7, 162.2, 161.3, 155.7, 138.8, 138.7, 129.1, 116.9, 114.1, 49.8, 37.6, 36.6, 36.1, 28.5, 22.8. HRMS (ESI+): *m/z* calcd. for [C25H30 N8O + 2H]^2+^: 230.1350, found: 230.1344.

#### Methyl 3‐(7‐amino‐2‐methyl‐1,8‐naphthyridin‐4‐yl)‐2‐((7‐amino‐2‐methyl‐1,8‐naphthyridin‐4‐yl)methyl)propanoate (20)

A solution of **10** (56.2 mg, 0.21 mmol), potassium carbonate (30.4 mg, 0.22 mmol), potassium iodide (22.9 mg, 0.138 mmol), and Meldrum's acid (14.4 mg, 0.10 mmol) in DMF (2 mL) was stirred at room temperature overnight under Ar. The mixture was concentrated *in vacuo*. To the residue, anhydrous methanol (5 mL) was added, and the mixture was stirred at reflux condition under Ar for overnight. Methanol was evaporated *in vacuo* and the resulting crude product was purified by column chromatography on amino silica gel (using chloroform/methanol, 100/0 to 97/3 as eluent) to afford **20** (31.0 mg, 74 %). ^1^H NMR (600 MHz, CDCl_3_) δ 7.72 (d, *J*=9.0 Hz, 2H), 6.90 (s, 2H), 6.63 (d, *J*=8.4 Hz, 2H), 5.07 (s, 4H), 3.48 (s, 3H), 3.37‐3.33 (m, 2H), 3.06‐3.04 (m, 3H), 2.64 (s, 6H). ^13^C NMR (150 MHz, CDCl_3_) δ 174.4, 161.8, 159.2, 156.6, 145.1, 133.6, 119.7, 114.1, 111.3, 52.0, 47.9, 33.7, 25.4. HRMS (ESI+): *m/z* calcd. for [C23H24 N6O2 + H]^+^: 417.2040, found: 417.2027.

#### 3‐(7‐amino‐2‐methyl‐1,8‐naphthyridin‐4‐yl)‐2‐((7‐amino‐2‐methyl‐1,8‐naphthyridin‐4‐yl)methyl)‐*N*‐(3‐aminopropyl)propenamide (ANP55)

A solution of **20** (31.0 mg, 0.07 mmol) in 1,3‐diaminopropane (3 mL) was stirred at 100 °C overnight under Ar. After the mixture was concentrated *in vacuo*, the residue was diluted with a small amount of water. The resulting crude product was purified by HPLC by using 0–30 % water‐acetonitrile (0.1 % AcOH) to afford **ANP55** (9.3 mg, 29 %). ^1^H NMR (600 MHz, CD_3_OD) δ 8.12 (d, *J*=9.6 Hz, 2H), 7.00 (s, 2H), 6.83 (d, *J*=9.0 Hz, 2H), 3.35 (dd, *J*=13.6, 10.5 Hz, 2H), 3.26 (dd, *J*=13.6, 5.0 Hz, 2H), 2.91‐2.89 (m, 1H), 2.81 (t, *J*=6.9 Hz, 2H), 2.56 (s, 6H), 2.35 (t, *J*=7.2 Hz, 2H), 1.29‐1.25 (m, 2H). ^13^C NMR (150 MHz, CD_3_OD) δ 175.8, 162.2, 161.8, 157.4, 148.7, 135.5, 120.4, 115.3, 113.5, 51.0, 37.7, 36.7, 35.3, 28.3, 24.6. HRMS (ESI+): *m/z* calcd. for [C25H30 N8O + H]^+^: 459.2622, found: 459.2608.

#### Methyl 3‐(2‐amino‐7‐methyl‐1,8‐naphthyridin‐4‐yl)‐2‐((2‐amino‐7‐methyl‐1,8‐naphthyridin‐4‐yl)methyl)propanoate (22)

**16** (35 mg, 0.14 mmol) was dissolved in DMF (3 mL) and Meldrum's acid (10.6 mg, 0.07 mmol), potassium iodide (15.2 mg, 0.09 mmol), and potassium carbonate (21.4 mg, 0.15 mmol) were added. The reaction mixture was stirred for overnight at room temperature under Ar, then the solvent was evaporated under reduced pressure. The resulting crude **21** was dissolved in methanol (2 mL) and stirred at reflux for 6 h under Ar atmosphere. The solvent was removed under reduced pressure and the resulting crude product was purified by column chromatography on amino silica gel (using mixture of chloroform and methanol as eluent) to afford **22** (6.0 mg, 21 %). ^1^H NMR (600 MHz, CD_3_OD) δ 8.00 (d, *J*=8.4 Hz, 2H), 7.05 (d, *J*=8.4 Hz, 2H), 6.65 (s, 2H), 3.41 (s, 3H), 3.32‐3.22 (m, 4H), 3.14‐3.11 (m, 1H), 2.58 (s, 6H). HRMS (ESI+): m/z cald. for [C_23_H_24_N_6_O_2_ + H]^+^: 417.2040, found: 417.2025.

#### 3‐(2‐amino‐7‐methyl‐1,8‐naphthyridin‐4‐yl)‐2‐((2‐amino‐7‐methyl‐1,8‐naphthyridin‐4‐yl)methyl)‐*N*‐(3‐aminopropyl)propenamide (ANP44)

**22** (5.9 mg, 0.014 mmol) was dissolved in 1,3‐diaminopropane (1.5 mL) and the solution was stirred at 100 °C for overnight under Ar. After the mixture was concentrated *in vacuo*, the residue was diluted with a small amount of water. The resulting crude product was purified by HPLC by using 0–30 % water‐acetonitrile (0.1 % AcOH) to afford **ANP44** (3.2 mg, 49 %). ^1^H NMR (600 MHz, D_2_O) δ 8.02 (d, *J*=7.8 Hz, 2H), 7.07 (d, *J*=7.8 Hz, 2H), 6.50 (s, 2H), 3.15 (dd, *J*=13.8, 6.0 Hz, 2H,), 3.06‐3.02 (m, 2H), 2.84‐2.82 (m, 1H), 2.63 (t, *J*=6.6 Hz, 2H), 2.46 (s, 6H), 2.25 (t, *J*=7.8 Hz, 2H), 1.15‐1.12 (m, 2H). ^13^C NMR (175 MHz, D_2_O) δ 174.8, 161.0, 160.0, 153.4, 148.2, 135.3, 119.0, 114.6, 113.1, 48.0, 36.4, 35.6, 33.5, 26.1, 22.8. HRMS (ESI+): m/z cald. for [C_25_H_30_N_8_O + 2H]^2+^: 230.1350, found: 230.1341.

#### Methyl 3‐(7‐amino‐1,8‐naphthyridin‐2‐yl)‐2‐((7‐amino‐2‐methyl‐1,8‐naphthyridin‐3‐yl)methyl)propanoate (24)

A solution of **23** ^[33]^ (39.7 mg, 0.10 mmol), potassium carbonate (13.8 mg, 0.10 mmol), and **8** (34.1 mg, 0.10 mmol) was stirred at room temperature for 4 h under Ar. The mixture was concentrated *in vacuo*, and the residue was dissolved in anhydrous methanol (6 mL). The solution was stirred under Ar at reflux for overnight. The solvent was evaporated *in vacuo*, and the residue was purified by column chromatography on amino silica gel (using chloroform/methanol, 98/2 as eluent) to afford **24** (19.1 mg, 47 %). ^1^H NMR (600 MHz, CD_3_OD) δ 7.90 (d, *J*=7.8 Hz, 1H), 7.84 (d, *J*=8.4 Hz, 1H), 7.79 (d, *J*=8.4 Hz, 1H), 7.75 (s, 1H), 7.08 (d, *J*=7.8 Hz, 1H), 6.80 (d, *J*=9.0 Hz, 1H), 6.76 (d, *J*=9.0 Hz, 1H), 3.63 (s, 1H), 3.48 (s, 3H), 3.31‐3.28 (m, 1H), 3.15‐3.08 (m, 2H), 2.98 (dd, *J*=14.4, 6.6 Hz, 1H), 2.56 (s, 3H). ^13^C NMR (150 MHz, CD_3_OD) δ 176.6, 162.8, 162.5, 162.2, 161.3, 157.3, 156.0, 139.1, 138.6, 138.5, 138.3, 128.7, 119.4, 117.1, 116.9, 114.0, 113.8, 52.2, 47.4, 41.8, 35.6, 22.7. HRMS (ESI+): *m/z* calcd. for [C22H22 N6O2 + H]^+^:403.1883, found: 403.1872.

#### 3‐(7‐amino‐1,8‐naphthyridin‐2‐yl)‐2‐((7‐amino‐2‐methyl‐1,8‐naphthyridin‐3‐yl)methyl)‐*N*‐(3‐aminopropyl)propenamide (ANP67)

**24** (19.0 mg, 0.047 mmol) was dissolved in 1,3‐diaminopropane (2.0 mL) and the solution was stirred at 100 °C for overnight under Ar. The excess amount of 1,3‐diaminopropane was evaporated under reduced pressure and the resulting crude product was purified by HPLC using 0–30 % water‐acetonitrile (0.1 % AcOH) to afford **ANP67** (6.7 mg, 32 %). ^1^H NMR (600 MHz, CD_3_OD) δ 7.97 (d, *J*=8.4 Hz, 1H), 7.90 (d, *J*=9.0 Hz, 1H), 7.83 (d, *J*=9.0 Hz, 1H), 7.79 (s, 1H), 7.14 (d, *J*=8.4 Hz, 1H), 6.83 (d, *J*=9.0 Hz, 1H), 6.80 (d, *J*=8.4 Hz, 1H), 3.29‐3.27 (m, 1H), 3.21‐3.17 (m, 1H), 3.14‐3.09 (m, 3H), 3.00 (dd, *J*=13.5, 4.5 Hz, 1H), 2.92‐2.88 (m, 1H), 2.69 (t, *J*=6.9 Hz, 2H), 2.62 (s, 3H), 1.62‐1.57 (m, 1H), 1.46‐1.41 (m, 1H). ^13^C NMR (150 MHz, CD_3_OD) δ 177.4, 163.2, 162.5, 162.2, 161.4, 157.3, 155.9, 139.3, 139.1, 138.7, 138.5, 129.1, 119.7, 117.1, 116.9, 114.2, 114.0, 62.3, 42.4, 38.1, 36.8, 35.9, 28.7, 22.8. HRMS (ESI+): *m/z* calcd. for [C24H28 N8O+2H]^2+^:223.1272, found: 223.1263.

#### Methyl 3‐(7‐amino‐1,8‐naphthyridin‐2‐yl)‐2‐((7‐amino‐2‐methyl‐1,8‐naphthyridin‐4‐yl)methyl)propanoate (25)

A solution of **23** (39.7 mg, 0.10 mmol), potassium carbonate (13.8 mg, 0.10 mmol), potassium iodide (16.6 mg, 0.10 mmol) and **10** (26.6 mg, 0.10 mmol) were stirred at room temperature for overnight under Ar. The mixture was evaporated *in vacuo*, and the residue was dissolved in anhydrous methanol (5 mL) and the mixture was stirred at reflux for overnight under Ar. The solvent was evaporated *in vacuo*, and the residue was purified by column chromatography on amino silica gel (using chloroform/methanol, 98/2 as eluent) to afford **25** (16.1 mg, 40 %). ^1^H NMR (600 MHz, CDCl_3_) δ 8.10 (d, *J*=8.4 Hz, 1H), 7.79 (d, *J*=7.8 Hz, 1H), 7.75 (d, *J* =9.0 Hz, 1H), 7.00 (d, *J*=7.8 Hz, 1H), 6.87 (s, 1H), 6.72 (d, *J*=9.0 Hz, 1H), 6.69 (d, *J*=9.0 Hz, 1H), 5.79 (s, 2H), 5.46 (s, 2H), 3.68‐3.64 (m, 1H), 3.48 (s, 3H), 3.34 (dd, *J*=14.4 Hz, 7.4 Hz, 1H), 3.27 (dd, *J*=13.7, 8.9 Hz, 1H), 3.17‐3.09 (m, 2H), 2.57 (s, 3H). ^13^C NMR (150 MHz, CDCl_3_) δ 175.3, 161.5, 161.2, 160.1, 159.5, 156.4, 156.3, 146.0, 138.0, 136.6, 134.4, 119.7, 118.6, 115.8, 114.4, 112.4, 111.6, 51.7, 45.7, 40.6, 33.9, 25.2. HRMS (ESI+): *m/z* calcd. for [C22H22 N6O2 + 2H]^2+^: 202.0981, found: 202.0974.

#### 3‐(7‐amino‐1,8‐naphthyridin‐2‐yl)‐2‐((7‐amino‐2‐methyl‐1,8‐naphthyridin‐4‐yl)methyl)‐*N*‐(3‐aminopropyl)propenamide (ANP57)

**25** (16.0 mg, 0.04 mmol) was dissolved in 1,3‐diaminopropane (2.0 mL) and the solution was stirred at 100 °C for overnight under Ar. The excess amount of 1,3‐diaminopropane was evaporated under reduced pressure and the resulting crude product was purified by HPLC by using 0–30 % water‐acetonitrile (0.1 % AcOH) to afford **ANP57** (5.4 mg, 30 %). ^1^H NMR (600 MHz, CD_3_OD) δ 8.18 (d, *J*=9.6 Hz, 1H), 7.98 (d, *J*=8.4 Hz, 1H), 7.91 (d, *J*=8.4 Hz, 1H), 7.13 (d, *J*=8.4 Hz, 1H), 7.00 (s, 1H), 6.85‐6.83 (m, 2H), 3.29‐3.14 (m, 5H; merged with CD_3_OD signal), 3.08‐3.03 (m, 1H), 2.92‐2.88 (m, 1H), 2.69‐2.63 (m, 2H), 2.56 (s, 3H), 1.59‐1.55 (m, 1H), 1.43‐1.39 (m, 1H). ^13^C NMR (150 MHz, CD_3_OD) δ 175.7, 161.6, 161.1, 160.8, 160.4, 156.3, 156.0, 147.7, 138.0, 137.1, 134.4, 119.1, 118.3, 115.8, 114.1, 112.9, 112.1, 41.0, 36.7, 35.5, 33.7, 27.3, 23.2. HRMS (ESI+): *m/z* calcd. for [C24H28 N8O + H]^+^: 445.2465, found: 445.2458.

#### 3‐(7‐amino‐1,8‐naphthyridin‐2‐yl)‐2‐((2‐amino‐7‐methyl‐1,8‐naphthyridin‐4‐yl)methyl)‐N‐(3‐aminopropyl)propenamide (ANP47)

A solution of **23** (24.6 mg, 0.06 mmol), potassium carbonate (10.4 mg, 0.07 mmol), potassium iodide (10.3 mg, 0.06 mmol) and **16** (17.6 mg, 0.07 mmol) were stirred at room temperature for overnight under Ar. The mixture was evaporated *in vacuo*, and the residue was dissolved in anhydrous methanol (5 mL) and the mixture was stirred at reflux for overnight under Ar. The solvent was evaporated *in vacuo*, and the residue was purified by column chromatography on amino silica gel (using chloroform/methanol, 98/2 as eluent) to afford Methyl 3‐(7‐amino‐1,8‐naphthyridin‐2‐yl)‐2‐((2‐amino‐7‐methyl‐1,8‐naphthyridin‐4‐yl)methyl)propanoate (**26**) (ca. 9.3 mg), which was used for the next step without further purification.

**26** (ca. 9.3 mg) was dissolved in 1,3‐diaminopropane (0.7 mL) and the solution was stirred at 100 °C for overnight under Ar. The excess amount of 1,3‐diaminopropane was evaporated under reduced pressure and the resulting crude product was purified by HPLC by using 0–30 % water‐acetonitrile (0.1 % AcOH) to afford **ANP47** (2.4 mg, 9 %, 3 steps from **23**). ^1^H NMR (600 MHz, D_2_O) δ 7.95 (d, *J*=7.8 Hz, 1H), 7.77‐7.76 (m, 2H), 7.00 (d, *J*=7.8 Hz, 1H), 6.88 (d, *J*=7.8 Hz, 1H), 6.71 (d, *J*=9.0 Hz, 1H), 6.47 (s, 1H), 3.25‐3.20 (m, 1H), 3.05‐2.78 (m, 6H), 2.49‐2.42 (m, 2H), 2.39 (s, 3H), 1.38‐1.31 (m, 2H). HRMS (ESI+): m/z cald. for [C_24_H_28_N_8_O + H]^+^: 445.2465, found: 445.2451.

#### Potassium 5‐((7‐((methoxycarbonyl)amino)‐2‐methyl‐1,8‐naphthyridin‐4‐yl)methyl)‐2,2‐dimethyl‐4,6‐dioxo‐1,3‐dioxan‐5‐ide (27)

A solution of **10** (63.5 mg, 0.24 mmol), potassium carbonate (33.0 mg, 0.24 mmol), potassium iodide (19.8 mg, 0.12 mmol), and Meldrum's acid (37.9 mg, 0.26 mmol) in THF (5 mL) with 1 drop of water was stirred at room temperature for 24 h under Ar. The mixture was concentrated *in vacuo*. The residue was washed with methanol to remove inorganic salts and the filtrate was evaporated *in vacuo*. The residue was dissolved to methanol again and the insoluble material was filtered off. The filtrate was added to diethyl ether to form precipitate. The precipitate was filtered and dried *in vacuo* to afford **27** (57.1 mg, 58 %). ^1^H NMR (400 MHz, CD_3_OD) δ 8.80 (d, *J*=9.2 Hz, 1H), 8.14 (d, *J*=9.2 Hz, 1H), 7.31 (s, 1H), 3.90 (s, 2H), 3.81 (s, 3H), 2.65 (s, 3H), 1.54 (s, 6H). ^13^C NMR (150 MHz, CD_3_OD) δ 169.8, 163.4, 156.0, 155.8, 154.9, 153.4, 137.8, 122.4, 118.9, 113.1, 102.9, 74.8, 53.1, 27.4, 25.8, 25.1. HRMS (ESI+): *m/z* calcd. for [C_18_H_18_N_3_O_6_K + H]^+^: 412.0912, found: 412.0899.

#### 3‐(7‐amino‐2‐methyl‐1,8‐naphthyridin‐3‐yl)‐2‐((7‐amino‐2‐methyl‐1,8‐naphthyridin‐4‐yl)methyl)‐*N*‐(3‐aminopropyl)propenamide (ANP56)

A solution of **27** (41.1 mg, 0.10 mmol), potassium carbonate (13.8 mg, 0.10 mmol) and **8** (34.1 mg, 0.10 mmol) in dry DMF (3.4 mL) were stirred at room temperature for 22 h under Ar. The mixture was concentrated *in vacuo*. To the residue, anhydrous methanol (5 mL) was added and stirred at reflux for overnight under Ar. Methanol was evaporated *in vacuo*. The residue was purified by column chromatography on amino silica gel (using chloroform/methanol, 99/1 as eluent) to afford Methyl 3‐(7‐amino‐2‐methyl‐1,8‐naphthyridin‐3‐yl)‐2‐((7‐amino‐2‐methyl‐1,8‐naphthyridin‐4‐yl)methyl) propanoate (**28**) (ca. 21.2 mg), which was used for the next step without further purification.

**28** (ca 21.2 mg) was dissolved in 1,3‐diaminopropane (3.5 mL) and the solution was stirred at 100 °C for 18 h under Ar. The excess amount of 1,3‐diaminopropane was evaporated under reduced pressure and the resulting crude product was purified by HPLC by using 0–30 % water‐acetonitrile (0.1 % AcOH) to afford **ANP56** (3.2 mg, 7 %, 3 steps from **27**). ^1^H NMR (600 MHz, CD_3_OD) δ 8.17 (d, *J*=9.6 Hz, 1H), 7.86 (d, *J*=8.4 Hz, 1H), 7.82 (s, 1H), 7.05 (s, 1H), 6.87 (d, *J*=9.0 Hz, 1H), 6.82 (d, *J*=9.0 Hz, 1H), 3.37‐3.28 (m, 2H; merged with CD_3_OD signal), 3.19‐3.15 (m, 1H), 3.08 (dd, J=13.9 Hz, 5.0 Hz, 1H), 2.94‐2.90 (m, 1H), 2.87‐2.82 (m, 2H), 2.65 (s, 3H), 2.58 (s, 3H), 2.41‐2.33 (m, 2H), 1.34‐1.28 (m, 2H). ^13^C NMR (100 MHz, CD_3_OD) δ 176.2, 162.3, 162.2, 161.7, 161.2, 157.3, 155.9, 149.1, 138.8, 138.7, 135.5, 128.8, 120.4, 116.9, 115.3, 114.1, 113.6, 50.4, 37.7, 36.6, 36.2, 35.2, 28.4, 22.8. HRMS (ESI+): *m/z* calcd. for [C25H30 N8O + 2H]^2+^: 230.1350, found: 230.1341.

## Conflict of Interests

The authors declare no conflict of interest.

1

## Data Availability

The data that support the findings of this study are available from the corresponding author upon reasonable request.
